# miR-103 inhibits proliferation and sensitizes hemopoietic tumor cells for glucocorticoid-induced apoptosis

**DOI:** 10.18632/oncotarget.13447

**Published:** 2016-11-18

**Authors:** Shlomit Kfir-Erenfeld, Noa Haggiag, Moshe Biton, Polina Stepensky, Nathalie Assayag-Asherie, Eitan Yefenof

**Affiliations:** ^1^ The Lautenberg Center for Immunology and Cancer Research, IMRIC, The Hebrew University-Hadassah Medical School, Jerusalem, Israel; ^2^ Pediatric Hemato-Oncology and Bone Marrow Transplantation Department, Hadassah-Hebrew University Medical Center, Jerusalem, Israel

**Keywords:** glucocorticoid, miR-103, leukemia, apoptosis, proliferation

## Abstract

Glucocorticoid (GC) hormones are an important ingredient of leukemia therapy since they are potent inducers of lymphoid cell apoptosis. However, the development of GC resistance remains an obstacle in GC-based treatment. In the present investigation we found that miR-103 is upregulated in GC-sensitive leukemia cells treated by the hormone. Transfection of GC resistant cells with miR-103 sensitized them to GC induced apoptosis (GCIA), while miR-103 sponging of GC sensitive cells rendered them partially resistant. miR-103 reduced the expression of cyclin dependent kinase (CDK2) and its cyclin E1 target, thereby leading to inhibition of cellular proliferation. miR-103 is encoded within the fifth intron of *PANK3* gene. We demonstrate that the GC receptor (GR) upregulates miR-103 by direct interaction with GC response element (GRE) in the *PANK3* enhancer. Consequently, miR-103 targets the c-Myc activators c-Myb and DVL1, thereby reducing c-Myc expression. Since c-Myc is a transcription factor of the miR-17~92a poly-cistron, all six miRNAs of the latter are also downregulated. Of these, miR-18a and miR-20a are involved in GCIA, as they target GR and BIM, respectively. Consequently, GR and BIM expression are elevated, thus advancing GCIA. Altogether, this study highlights miR-103 as a useful prognostic biomarker and drug for leukemia management in the future.

## INTRODUCTION

Glucocorticoids (GCs) are widely used in the therapy of lymphoma and leukemia due to their ability to induce apoptosis and proliferation arrest of lymphoid cells. Prednisone (PRED) and Dexamethasone (Dex), both synthetic GC analogues, are major components in the therapy of many immunological disorders, including hematopoietic malignancies. Acute lymphoblastic leukemia (ALL) is a malignant disease characterized by replacement of normal hematopoietic cells in the bone marrow by undifferentiated and highly proliferative lymphoid precursors of T or B origin (referred as T-ALL or B-ALL, respectively). Newly diagnosed ALL patients are initially treated with PRED, as a sole medication, for seven days. About 75% of ALL patients respond well to PRED treatment and are classified as PRED good responders (PGRs). The remaining PRED resistant patients are defined as PRED poor responders (PPRs) [1, 2]. While PGR patients receive standard therapeutic regimen, PPR patients are subjected to a high risk (HR) protocol. Since the HR protocol implicates highly toxic side effects, it is of great clinical interest to evaluate the real necessity of such treatment. To this end, it is imperative to study the molecular basis of GC-mediated therapy and the mode by which cells resist PRED treatment, in order to improve the therapeutic outcome.

In the absence of GC, the GC receptor (GR) is sequestered in the cytosol by a heteromeric protein complex [3]. Upon GC binding, the complex dissociates to enable GR activation and nuclear translocation [4]. In the nucleus, GR modulates expression of multiple genes by interaction with GC response elements (GRE) or by trans-regulation of other transcription factors [5–7]. Most of the GR regulated genes are unrelated to apoptosis [8].

Numerous studies were performed on the biochemical events leading to acquisition of GC apoptotic resistance. GR nuclear activities occur in both GC-sensitive and GC-resistant cells. However, the gene expression profile is distinguishable between such cells. Several apoptosis related genes were found to be regulated in GC-sensitive, but not resistant cells [8]. Of special note are the upregulation of the GR [9] and BIM [10], and the downregulation of c-Myc [11].

GR expression is auto-upregulated in GC-sensitive cells following GC treatment [9]. This feedback is enabled by a GRE located within the GR promoter [9, 12] and acts to amplify the apoptotic response [13]. Indeed, GC-resistant cells downregulate the GR upon GC treatment [14]. However, many GC-resistant cells express high basal levels of GR [15], indicating that GR expression is not the sole factor required for GC-induced apoptosis (GCIA), although a threshold GR level is mandatory to initiate GCIA.

Besides GR, the most important gene upregulated during GCIA is BIM [10]. BIM^−/−^ thymocytes and BIM knocked down lymphoma cells display resistance to GCIA [16, 17]. Therefore, in most ALL cells, GC-induced BIM upregulation is mandatory for advancing GCIA [18]. Indeed, ALL patients categorized into PGR subgroup upregulate BIM expression after 7 days of PRED monotherapy, while in ALL patients belonging to PPR category BIM expression is downregulated or remains unchanged. Hence, BIM is a good prognostic biomarker for PRED response in ALL [19, 20].

Upon GC stimulation, c-Myc is downregulated in GC-sensitive, but not in GC-resistant cells [11, 21]. In some B cell lymphomas c-Myc is deregulated as a consequence of a cMyc/Ig chromosomal translocation [22], while in many T-ALLs, c-Myc and its target genes are directly upregulated by IC-NOTCH [23]. Hence, GC-induced c-Myc downregulation may offset the tumorigenic phenotype of malignant lymphoid cells.

It has been discovered that GR regulates microRNA expression in lymphoid cells [24–29]. For instance, GC enhances apoptosis by modulating the expression of miR-15~16 [24]. Indeed, overexpression of miR-15~16 in T-ALL increased GCIA while its silencing decreased it [24]. In addition, GC downregulates the miR-17~92a poly-cistron (containing six mi-RNA: miR-17, miR-18a, miR-19a, miR-20a, miR-19b, and miR-92a) [25, 26]. miR-17~92a is upregulated in T-ALL and promotes leukemogenesis [30]. It was demonstrated that GCs upregulate BIM expression through downregulation of miR-17~92a [26]. Indeed, BIM is downregulated by miR-17~92a [31], mostly by miR-17, miR-19 and miR-92a [30, 32, 33]. c-Myc, which is downregulated by GC, is a transcription factor of miR-17~92a [34], and deletion of miR-17~92a represses c-Myc-induced oncogenesis [35].

By using deep sequencing analysis to compare GC-regulated miRNAs in GC-sensitive ALL cells, we found that miR-103 stands out as the most significant miRNA that is upregulated during GCIA. Furthermore, miR-103 overexpression confers sensitivity to GCIA on otherwise GC-resistant cells. In the present study, we outline the mode by which miR-103 is involved in GCIA and cell cycle arrest. We suggest that miR-103 plays a pivotal role in GCIA and that its upregulation sensitizes GC resistant cells to GCIA.

## RESULTS

### Glucocorticoid hormones in the treatment of acute lymphoid leukemia (ALL)

**Figure 1:**
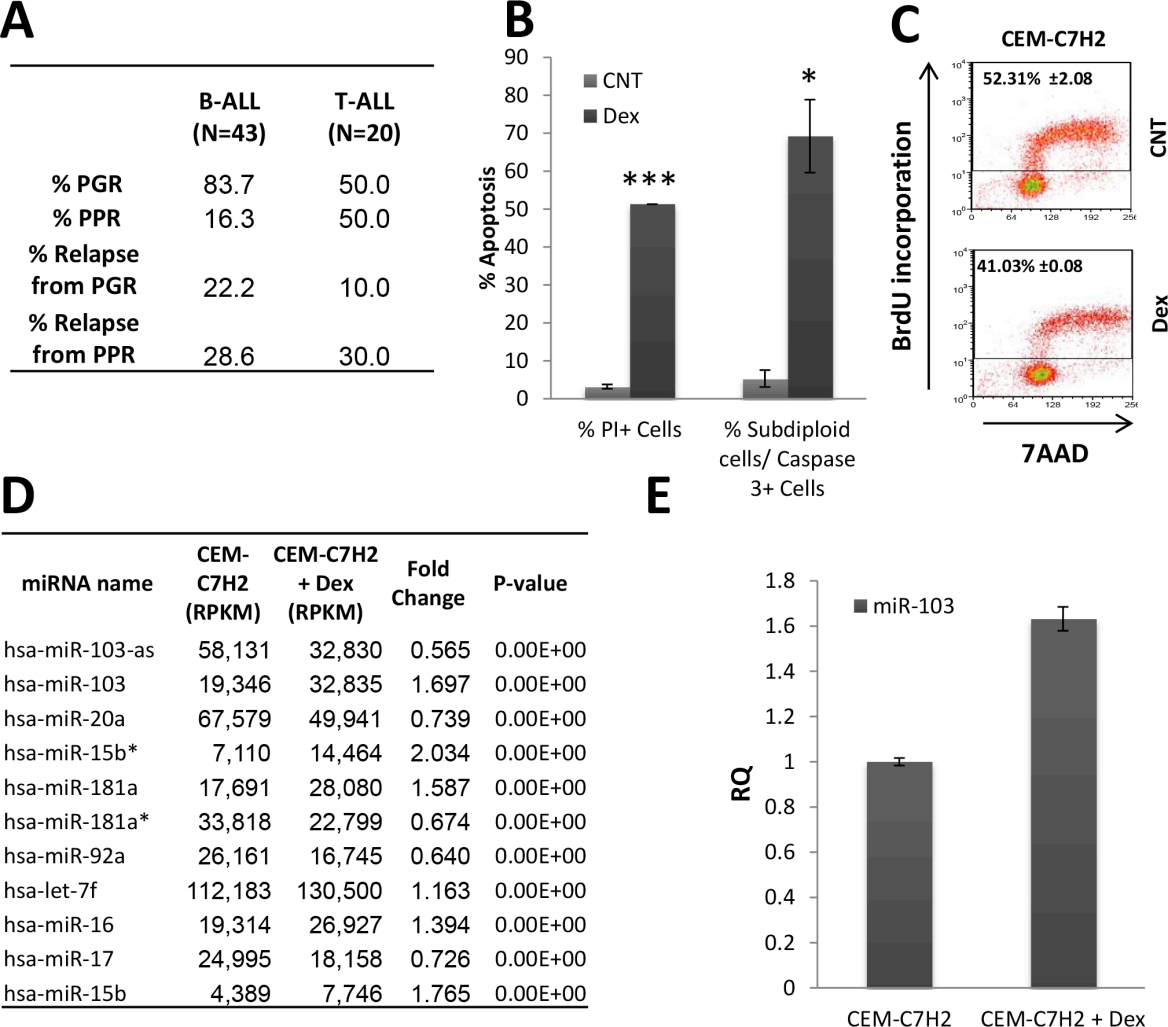
Relevance of miR-103 in ALL (**A**) Response of ALL patients to prednisone-treatment. A cohort of B- and T-ALL patients (*N* = 43 and 20, respectively) were monitored following prednisone-treatment. (PPR; absolute blast count in peripheral blood ≥ 1000/μl). (**B**) and (**C**) Response of the sensitive CEM-C7H2 cells to Dex-treatment. (B) Dex-induced apoptosis. CEM-C7H2 T-ALL cells were untreated or 100nM Dex-treated for 72 hours. Cells were stained with propidium iodide (PI) for PI positive test or fixed and stained for both PI and Caspase-3 antibody. The percent of PI-positive and Caspase-3-positive cells were analyzed by flow cytometry. (C) Dex inhibits cell proliferation. CEM-C7H2 were untreated or Dex-treated for 24 hours, and further labeled with BrdU (1 hr), fixed and stained for both anti-BrdU antibody and 7AAD and analyzed by flow cytometry. The percent of BrdU incorporation is indicated in the corresponding panels. (**D**) miRNAs modulation in the sensitive CEM-C7H2 cells upon Dex-treatment. CEM-C7H2 cells were untreated or Dex-treated for 24 hrs and total RNA was extracted and sent for deep sequencing analysis. Most significantly affected miRNAs are indicated in the table. (**E**) miR-103 expression in CEM-C7H2 following Dex-treatment. CEM-C7H2 cells were untreated or Dex-treated for 24 hrs. RNA was extracted and miR-103 was quantified by qRT-PCR analysis.

The clinical management of ALL widely relies on GC-based regimen. Indeed, Figure 1A indicates that most ALL patients (50% of remission in the case of T-ALL, *N* = 43; 83% in the case of B-ALL, *N* = 20) are good responders to Prednisone (PRED) treatment (PRED Good Response, PGR; absolute blast count in peripheral blood ≤ 1000/μl after 7 days of PRED administration). However, 10% and 22% of PGR B-ALL and T-ALL patients, respectively, relapse. In addition, half of T-ALL and 16.3% of B-ALL d patients are poor responders to PRED treatment (PRED Poor Response, PPR; absolute blast count in peripheral blood ≥ 1000/μl after 7 days of PRED administration). The relapse rate of PPR ALL patients is higher than PGR ALL patients with approximately 30% to both B and T- ALL. Therefore, the PRED effect is one of the most important prognostic markers according to AIEOP-BFM ALL 2009 protocol [1, 2]. Consequently, after 7-days of PRED treatment, PPR patients are reassigned to high-risk protocols including aggressive chemotherapies and/or BM-transplantation. Hence, the effectiveness of GC treatment in ALL is limited, since some patients are less responsive to GC-based therapy, and others acquire resistance along the treatment. Furthermore, PGR ALL patients relapse, albeit with a lower rate, indicating that prognosis is estimated with insufficient accuracy and that applying high risk regimen might well avoid relapse in some patients. Therefore, it is of a major interest to get a profound understanding of the mechanisms involved in GC-induced apoptosis (GCIA).

We analyzed the effect of Dex on apoptosis of the GC-sensitive CEM-C7H2 cell. Flow cytometry analysis, showed that Dex induces apoptosis in 51.3% of the cells as determined by propidium iodide (PI) staining, or 69.2 ± 9.6% based on the percent of the sub-diploid Caspase-3-positive cells (Figure 1B). Additionally, BrdU incorporation analysis indicates that CEM-C7H2 cells display a significant decrease in their proliferation rate following Dex treatment (Figure 1C). To gain an insight into the molecular pathways regulating GCIA and GC-induced proliferation inhibition, CEM-C7H2 cells treated with Dex or untreated, were subjected to deep sequencing of small RNAs (Supplementary Table S1). This analysis revealed eleven miRNAs that were most significantly regulated by Dex in the sensitive CEM-C7H2 cells (Figure 1D). None of these miRNAs were significantly modulated in Dex-treated GC-resistant MOLT-4 cells (Supplementary Table S2). As miR-103 stood out as the most significant Dex- modulated miRNA, we decided to focus on its involvement in both proliferation and apoptosis. miR-103 real time PCR (qRT-PCR) analysis of Dex-treated CEM-C7H2 (Figure 1E) validated the deep sequencing data (Figure 1D), marking miR-103 as significantly modulated upon GC-treatment.

### miR-103 inhibits cellular proliferation

**Figure 2:**
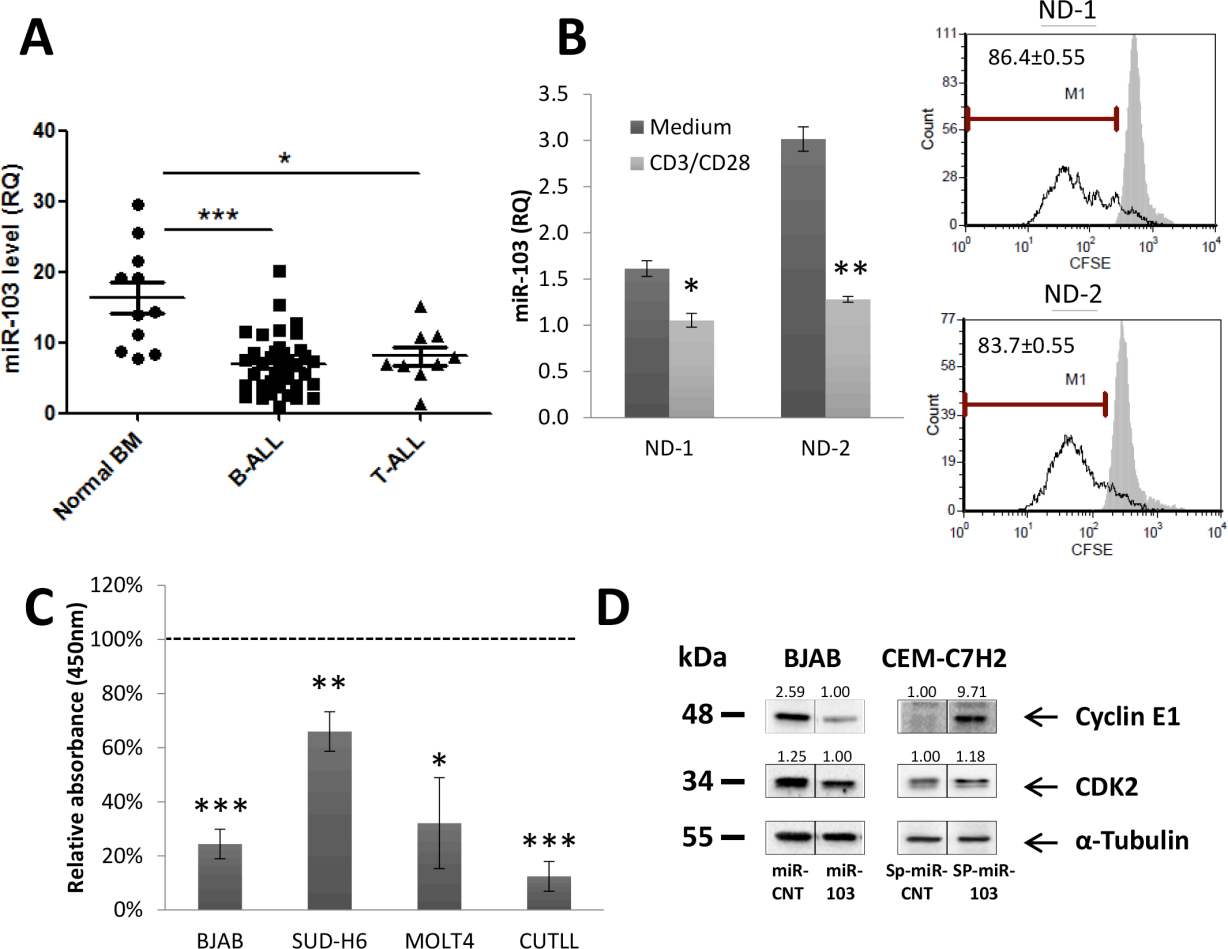
miR-103 upregulation induces inhibition of cell proliferation (**A**) miR-103 downregulation in B- and T-ALL bone-marrow (BM)-isolated mononuclear cells. Normal BM samples, B- and T-ALL BM (*N* = 11, 38 and 9, respectively) were subjected to Ficoll gradient, RNA extraction and qRT-PCR analysis of miR-103. (**B**) miR-103 downregulation in peripheral blood mononuclear cells from normal donors (ND) stimulated to proliferation for 4 days with anti-CD3/CD28 or unstimulated. CFSE dilution in proliferative CD3-positive cells is shown in the right panel. (**C**) miR-103 overexpressing -CUTLL, -MOLT-4, -SUD-H6 and -BJAB cells were analyzed for BrdU incorporation by ELISA, and compared with miR-control (CNT)-transfected cells. BrdU values in miR-103 clones were normalized according to the miR-CNT values obtained in each corresponding cell line. (**D**) miR-103 overexpressing BJAB cells or sponged-miR-103 CEM-C7H2 cell lysates were subjected to Western blot analysis using anti-Cyclin E1 and CDK2 antibodies.

We compared the basal expression of miR-103 in leukemia patients and healthy counterparts. To this end, the level of miR-103 was determined in bone marrow-derived mononuclear cells (MCs) from healthy donors and ALL patients. We observed that the miR-103 level is significantly downregulated in ALL MCs compared with normal bone marrow-derived MCs (Figure 2A). Since tumorigenesis is associated with high proliferative state of the cancer cells, we asked whether miR-103 decrease in ALL MCs can be related to cellular proliferation. To answer this question, we analyzed miR-103 expression in peripheral blood mononuclear cells (PBMCs) from normal donors (ND) stained with CFSE and stimulated with anti- CD3 and anti-CD28 antibodies for 4 days. Figure 2B shows that CFSE dilution (i.e., > 90% proliferative T cells, data not shown) is associated with a decrease in miR-103 level. To confirm that inhibition of cellular proliferation is miR-103-dependent, CUTLL, MOLT-4, SUD-H6 and BJAB cells were either infected with miR-103 or miR-CNT (CNT) vectors (Figure 2C) and further assessed for BrdU incorporation by an ELISA assay. Figure 2C shows that miR-103 expression in miR-103-transfected cell lines induces a significant reduction in their proliferative rate. Interestingly, analysis of the basal level of miR-103 in different human organs reveals that there is an inverse correlation between the level of miR-103 and the proliferative state of the organ (Supplementary Figure S1A). Again, this observation supports the concept that miR-103 is associated with cellular proliferation. Indeed, it has been reported that GC also induces cell cycle arrest (Figure 1C) [36, 37]. Along this line, we found that miR-103 also reduced the total cell number in the culture, which was further decreased by Dex but abolished when GR was inhibited by RU486 (Supplementary Figure S1B). Screening of the literature points out at cyclin E1 and its positive regulator CDK2, as essential for the G1 −> S transition. These proteins are direct targets of miR-103 [38], and were found to be downregulated by miR-103 overexpression in our study as well (Figure 2D and Supplementary Figure S1C–S1E). Vice versa, when miR-103 is sponged (with sponge vector described in Supplementary Figure S1F), there is a marked induction of both cyclin E1 and CDK2 expression (Figure 2D).

### miR-103 regulation upon Dex stimulation

**Figure 3:**
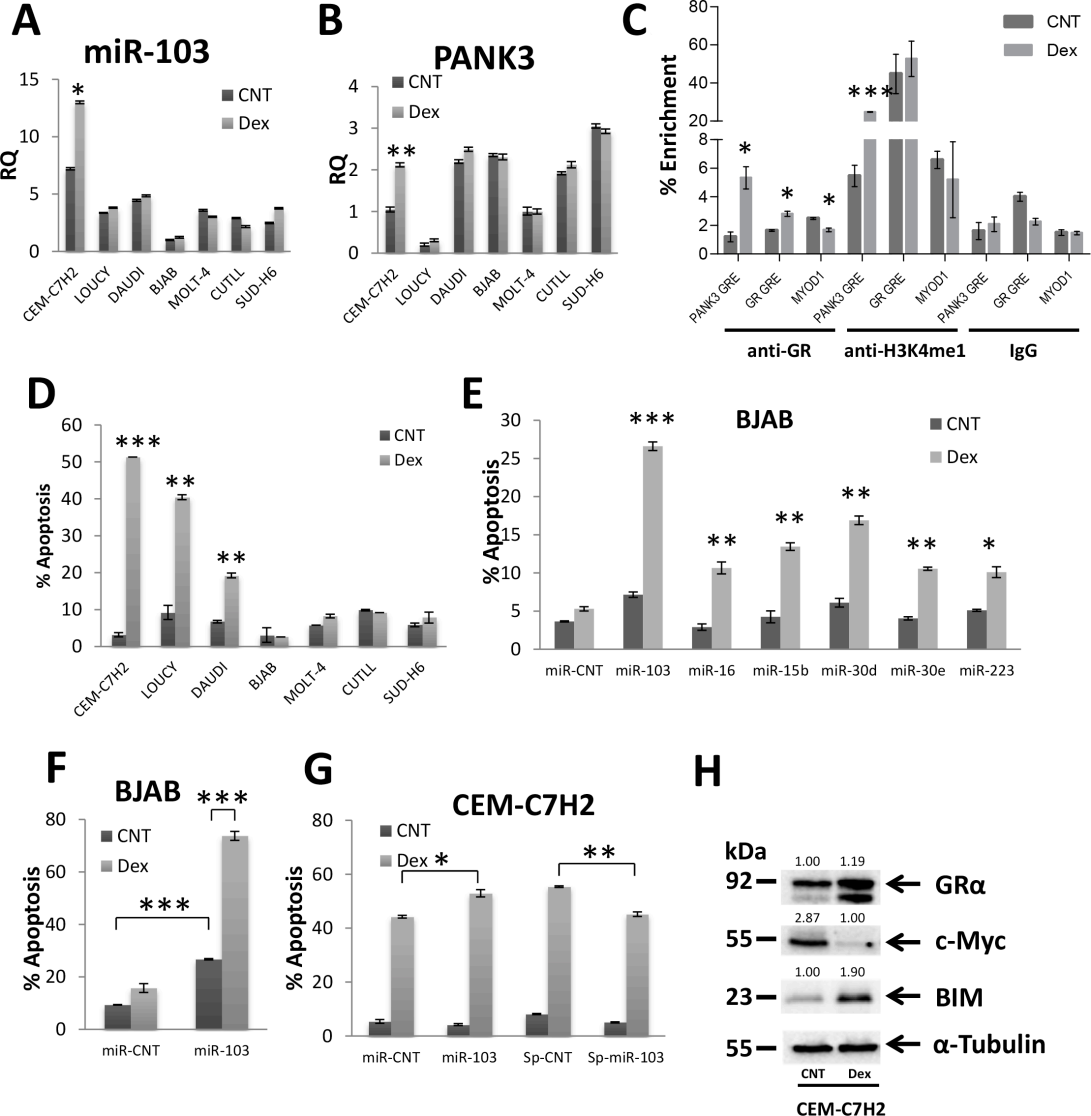
miR-103 sensitizes to GCIA and is regulated by binding of GR to PANK3 enhancer (**A**) and (**B**) Relative quantitation (RQ) of miR-103 and *PANK3* levels in GC-sensitive and – resistant cells. CEM-C7H2, LOUCY, DAUDI, BJAB, MOLT-4, CUTLL, SUD-H6 were untreated or Dex-treated for 24 hrs, RNA was extracted and analyzed for miR-103 (A) and *PANK3* (B) by qRT-PCR. (**C**) GR binds to GRE within *PANK3* promotor. Chromatin of untreated and Dex treated CEM-C7H2 cells was immunoprecipitated with anti-GRα, controls anti-H3K4me1 (positive) or anti-IgG (negative) antibodies. DNA was then subjected to qRT-PCR with primers of the PANK3-GRE, GR-GRE and MYOD1 promoters. Percent enrichment relative to input was measured. (**D**) Characterization of Dex-sensitive and -resistant cells. CEM-C7H2, LOUCY, DAUDI, BJAB, MOLT-4, CUTLL and SUD-H6 and were untreated or Dex-treated for 72 hrs and then analyzed for PI-staining by flow cytometry. The percent of PI-positive apoptotic cells is indicated. (**E**) and (**F**) miR-103 sensitizes GC-resistant BJAB cells to GC-induced apoptosis. BJAB cells were transfected with various overexpression or sponge plasmids (E) and with miR-103 overexpression plasmid (F). Transfectant cells were untreated or treated with Dex for 72 hrs and the percentage of apoptotic cells was analyzed by flow cytometry of PI stained cells. (**G**) GC-sensitive CEM-C7H2 cells were transfected with miR-103 overexpressing or sponge plasmids and treated with Dex for 72 hrs. The percentage of PI-positive apoptotic cells was determined by flow cytometry. (**H**) GC-sensitive CEM-C7H2 cells respond to GCIA by upregulation of GR and BIM, and downregulation of c-Myc. CEM-C7H2 were untreated or treated with Dex for 24 hrs, and cell lysates were subjected to Western blot analysis using anti-GR, -c-Myc, -BIM, -Tubulin antibodies.

miR-103 resides within the fifth intron of the *PANK3* gene (Supplementary Figure S1G) [39]. In order to investigate whether miR-103 and *PANK3* are co-regulated, we first analyzed the relative quantification (RQ) of these two genes in untreated or Dex-treated cells. As shown in Figure 3A–3B, there is no correlation between basal miR-103 and *PANK3* levels. However, following Dex treatment, miR-103 and *PANK3* were both upregulated in CEM-C7H2 but remained unchanged in other cells (Figure 3A and 3B). Moreover, inhibition of GR by its inhibitor RU486, blocked both miR-103 and *PANK3* upregulation (Supplementary Figure S1H).

In analyzing the *PANK3* promoter, we detected a GRE sequence ~10,000 bp upstream to its coding region. This domain (TGTGCAAACTATTCTT) is located at chr5:168017288-168017303 and is compatible with a consensus GRE [5, 6]. We have therefore applied chromatin immunoprecipitation (ChIP) assay on CEM-C7H2 cells untreated or treated with Dex. Using an anti-GR antibody, we found that the *PANK3*-GRE was enriched by approximately 5% in Dex-treated compared with untreated cells (Figure 3C), indicating that upon activation, GR binds to GRE within the *PANK3* promoter. Furthermore, *PANK3*-GRE immunoprecipitated by anti-H3K4me1 was enriched nearly fivefold in Dex-treated cells (Figure 3C), suggesting that *PANK3*-GRE is an enhancer. No Dex-induced *PANK3*-GRE enrichment by control IgG antibody was observed (Figure 3C). As a positive control we used two *bona fide* GRE domains of the GR promoter [12]. Indeed, GR IP in Dex-treated cells was followed by enrichment of GR-GRE while the negative control MYOD1 was not enriched (Figure 3C).

### miR-103 confers GCIA

To evaluate the effect of miR-103 on GCIA, we assessed GC-sensitive and GC-resistant cell lines for apoptosis. To this end, CEM-C7H2, LOUCY, DAUDI, BJAB, MOLT-4, CUTLL, and SUD-H6 cell lines were treated with Dex for 72 hours. CEM-C7H2, LOUCY and DAUDI cells responded with apoptosis, while BJAB, MOLT-4, CUTLL, and SUD-H6 remained alive (Figure 3D). BJAB cells exhibit the lower percent of apoptosis upon Dex treatment (Figure 3D), and also display the lowest level of miR-103 expression (Figure 3A). For this reason, we chose to focus on BJAB cells for further analysis. To confirm that miR-103 plays an essential role in GCIA and is a Dex-regulated, we constructed overexpression and sponge plasmids (Supplementary Figure S1F) of the first sixteen Dex-regulated miRNAs in CEM-C7H2 (Supplementary Table S1) and searched for additional miRNAs that impose a GC-sensitive phenotype on otherwise GC-resistant cells. Indeed, transfection with miR-103 was particularly effective in conferring GC-sensitivity upon BJAB cells (Figure 3E). This parallels the outcome of the deep sequencing data, marking miR-103 as most significantly modulated upon Dex-stimulation (Figure 1D). Consequently, we tested the effect of miR-103 on GCIA by transfection with more concentrated miR-103 overexpression or sponge vectors (e.g., following ultra-centrifuge process). In some untreated cells, miR-103 overexpression had only a minor effect on apoptosis. However, when treated with Dex, resistant BJAB, MOLT-4, CUTLL, and SUD-H6 cells overexpressing miR-103 underwent significant apoptosis (Figure 3F and Supplementary Figure S2A–S2C). Moreover, sensitive CEM-C7H2, DAUDI and LOUCY cells overexpressing miR-103 became hyper-sensitive to GCIA (Figure 3G and Supplementary Figure S2D–S2E). Vice versa, when miR-103 was sponged in CEM-C7H2, LOUCY and DAUDI, a slight reduction in apoptosis was observed following treatment with Dex (Figure 3G and Supplementary Figure S2A–S2B). These data indicate that miR-103 plays a pivotal role in GCIA. The partial reduction in GCIA following miR-103 sponging can be attributed to potential redundant pathways with overlapping GCIA-related functions. This observation indicates that miR-103 may act as a tumor suppressor by both inhibiting cellular proliferation (Figure 2) and conferring sensitivity to GCIA (Figure 3).

To clarify the mode of action of miR-103, we analyzed its effect on consensus GC-affected proteins. To this end, we enumerated the expression of GR, BIM and c-Myc proteins upon Dex exposure. Western blot analysis (Figure 3H) confirmed that GR and BIM are upregulated in Dex-treated CEM-C7H2 GC-sensitive cell line, whereas c-Myc is downregulated.

### miR-103 downregulates c-Myc expression

**Figure 4:**
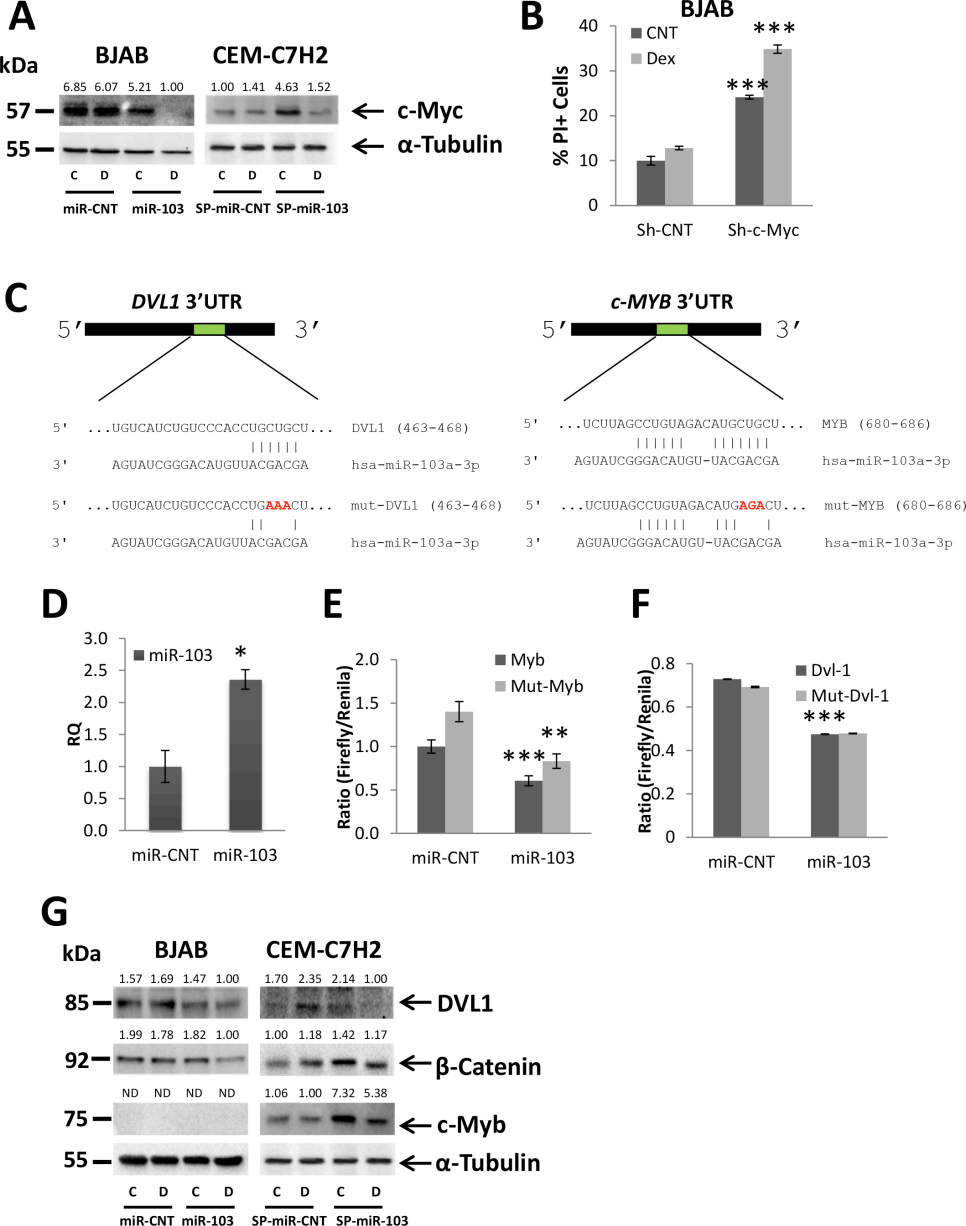
miR-103 downregulates c-Myc expression via inhibiting DVL1 and c-Myb (**A**) BJAB resistant and CEM-C7H2 sensitive cells were transfected with miR-103 overexpression or sponge plasmids, respectively. Cells were left untreated or treated with Dex for 24 hrs and subjected to Western blot analysis using anti-c-Myc antibody. (**B**) Percent of PI-stained BJAB cells transfected with Sh-c-Myc and treated with Dex for 72 hrs. (**C**) Schematic representation of the predicted binding sites for miR-103 in the 3′UTR of DVL1 and MYB. (**D**) qRT-PCR for the expression of miR-103 in 293T cells transfected with either a control miR (miR-CNT) or miR-103. (**E**) and (**F**) Wild type or mutated DVL-1 3′UTR (DVL-1 or mut-DVL-1, respectively) (F) and wild type or mutated MYB 3′UTR (MYB or mut-MYB, respectively) MYB 3′UTR (**G**) were fused to CDS of Firefly luciferase, and co-transfected with Renilla luciferase into 293T cells overexpressing miR-103 or miR-CNT. The luciferase activity was measured and normalized to Renilla activity. (G) Western blot analysis of Dex-treated BJAB and CEM-C7H2 using anti-DVL1, anti-β-Catenin and anti-c-Myb antibodies.

c-Myc, which is downregulated by GC in sensitive CEM-C7H2 and LOUCY (Supplementary Figure S3A) cells transfected with the miR-CNT vector but to a much lower extent in resistant BJAB (Figure 4A), CUTLL, SUD-H6, and MOLT-4 cells (Supplementary Figure S3A), was reduced by miR-103 overexpression. c-Myc was further downregulated following Dex treatment (Figure 4A and Supplementary Figure S3A). Moreover, sponging miR-103 in CEM-C7H2 cells, upregulated c-Myc expression (Figure 4A). However, upon Dex treatment, c-Myc expression was nullified (Figure 4A), explaining why GCIA of miR-103 sponged cells was only decreased but not abolished (Figure 3G). It seems that Dex treatment upregulates miR-103 to levels excessive of the sponge absorption capacity. In order to demonstrate the impact of c-Myc ablation on GCIA, BJAB cells were transfected with sh-RNA of c-Myc. This manipulation imitates the effect of miR-103 overexpression on GCIA (Figure 4B).

### miR-103 downregulates c-Myc by targeting DVL1 and c-Myb

c-Myc 3′UTR does not contain a seed sequence for miR-103. However, miR-103 was predicted (computational analysis using TargetScan and miRanda software) to target two potential c-Myc activators, c-Myb and DVL1. DVL1 is known to activate β-Catenin, which similarly to c-Myb, acts as a transcription factor of c-Myc [40, 41]. Figure 4C shows the predicted sites of miR-103 binding in the 3′UTRs of DVL1 and c-Myb, To investigate whether miR-103 directly targets both c-Myb and DVL1, we mutated three nucleotides in the predicted binding sites of miR-103 (Figure 4C), cloned both the mutated and wild type 3′UTR of DVL1 and c-Myb, respectively, downstream to a luciferase reporter gene, and assessed the luciferase activity in the presence of miR-103. To this end, miR- CNT or miR-103 overexpressing 293T cells (Figure 4D) were co-transfected with Firefly luciferase reporter PGL3 vector containing either the native 3′UTR of c-Myb and DVL1, or the mutated 3′UTR (as described in Figure 4C), together with a control Renilla luciferase pRL-CMV vector. Firefly luciferase activity was measured two days post-transfection and normalized to the activity of the control Renilla reporter. Figure 4E–4F indicates that miR-103 overexpression significantly decreases Firefly activity in 293T transfected with the wild type 3′UTRs of c-Myb and DVL1, respectively. Mutation in the seed sequence of c-Myb partially restored the luciferase activity (Figure 4E) while the mutated 3′UTR of DVL1 failed to do so (Figure 4F). Together, these findings indicate that miR-103 effectively targets the 3′UTRs of c-Myb and DVL1, respectively. The mutation in the DVL1 seed sequence was not efficient enough to restore luciferase activity. Alternatively, it may be possible that the mutation in the seed region generated a new sequence which effectively binds to unspecific miRNAs.

c-Myb is highly expressed in CEM-C7H2, LOUCY, CUTLL and MOLT-4 cells (Supplementary Figure S3B), but undergoes significant downregulation by Dex only in CEM-C7H2 and LOUCY sensitive cells (Supplementary Figure S3D). Note that c-Myb expression was not detected in BJAB and SUD-H6 resistant cell lines (Figure 4G and Supplementary Figure S3B). DVL1 is expressed in CEM-C7H2, LOUCY, BJAB, and SUD-H6 cells but is almost inexistent in CUTLL and MOLT-4. Following Dex exposure, both DVL1 and β-Catenin were downregulated in the sensitive CEM-C7H2 and LOUCY miR-CNT cells, but barely affected in the resistant cell lines (Supplementary Figure S3B).

We measured the level of these proteins in miR-103 overexpressing *vis-a-vis* sponged cells and found that both DVL1 and β-Catenin were downregulated by miR-103 in BJAB, SUD-H6 and LOUCY (in BJAB, β-Catenin is downregulated by miR-103 only with additional Dex treatment), and upregulated when the level of miR-103 was reduced by its sponge (Figure 4G and Supplementary Figure S3B). However, DVL1 was not detected in MOLT-4 and CUTLL and its expression in CEM-C7H2 was even upregulated when miR-103 was overexpressed (Supplementary Figure S3B). Likewise, the level of β-Catenin was unaffected by miR-103 in CEM-C7H2 and even upregulated in MOLT4 (Supplementary Figure S3B). By contrast, miR-103 downregulates c-Myb expression in CEM-C7H2, LOUCY, CUTLL and MOLT-4 T-ALL cells (Figure 4G and Supplementary Figure S3B). Moreover, upon miR-103 sponging, c-Myb expression is significantly increased (Figure 4G). However, c-Myb upregulation by miR-103 sponge is almost eliminated upon Dex induction (Figure 4G), explaining the low effect of miR-103 sponge on GCIA. We failed to detect c-Myb in B cell lymphomas, as this protein plays an essential role mostly in T, but less in B cell development (Supplementary Figure S3B) [42].

### miR-103 downregulates expression of miR-17~92a

**Figure 5:**
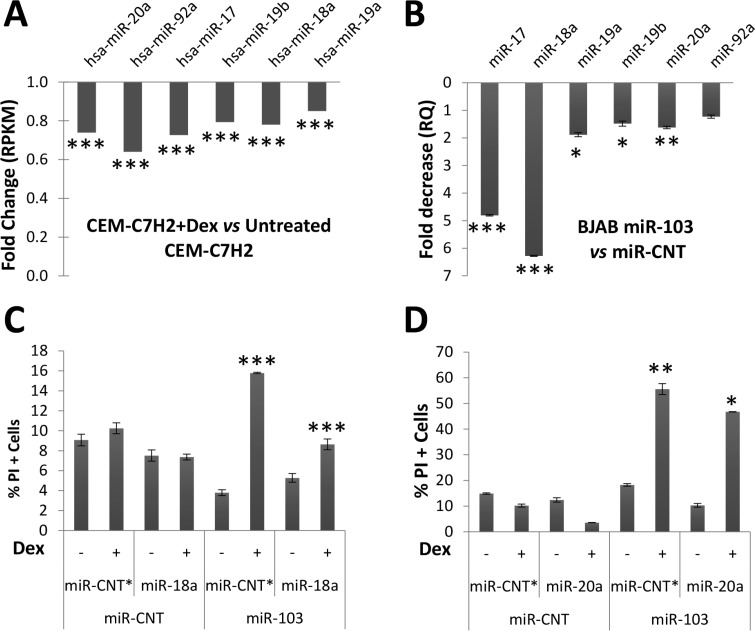
miR-103 downregulates miR-17~92a expression (**A**) Deep sequencing analysis of GC-sensitive CEM-C7H2 cells treated with Dex and compared with untreated corresponding cells. (**B**) Decreased RQ of miR-17~92a members measured in miR-103 overexpressing BJAB cells as compared with control cells. (**C**) and (**D**) miR-103 overexpressing BJAB cells were transfected with miR-18a (C) or miR-20a (D). The cells were then treated with Dex for 72 hrs and the percent of apoptotic cells was determined by PI staining.

c-Myc is a transcription factor of the oncogenic miR-17~92a multi-cistron [34]. Indeed, in CEM-C7H2 cells, which responded to GC with downregulation of c-Myc (Figure 3H), miR-17~92a was also downregulated following Dex treatment (Figure 5A) [25, 26]. However, in GC-resistant cells, both miR-17~92a and c-Myc were almost unaffected by Dex (Data not shown). When miR-103 was overexpressed in GC-resistant BJAB cells, a reduced expression of miR-17~92a was recorded (Figure 5B). To verify whether the downregulation of miR-17~92a is involved in miR-103 mediated GCIA, we transfected BJAB cells overexpressing miR-103 with plasmids containing each miRNA of miR-17~92a. Only miR-18a and miR-20a re-expression counteracted the miR-103-mediated GCIA (Figure 5C–5D and Supplementary Figure S3C–S3F).

### Suppression of miR-18a by miR-103 enables GR upregulation

**Figure 6:**
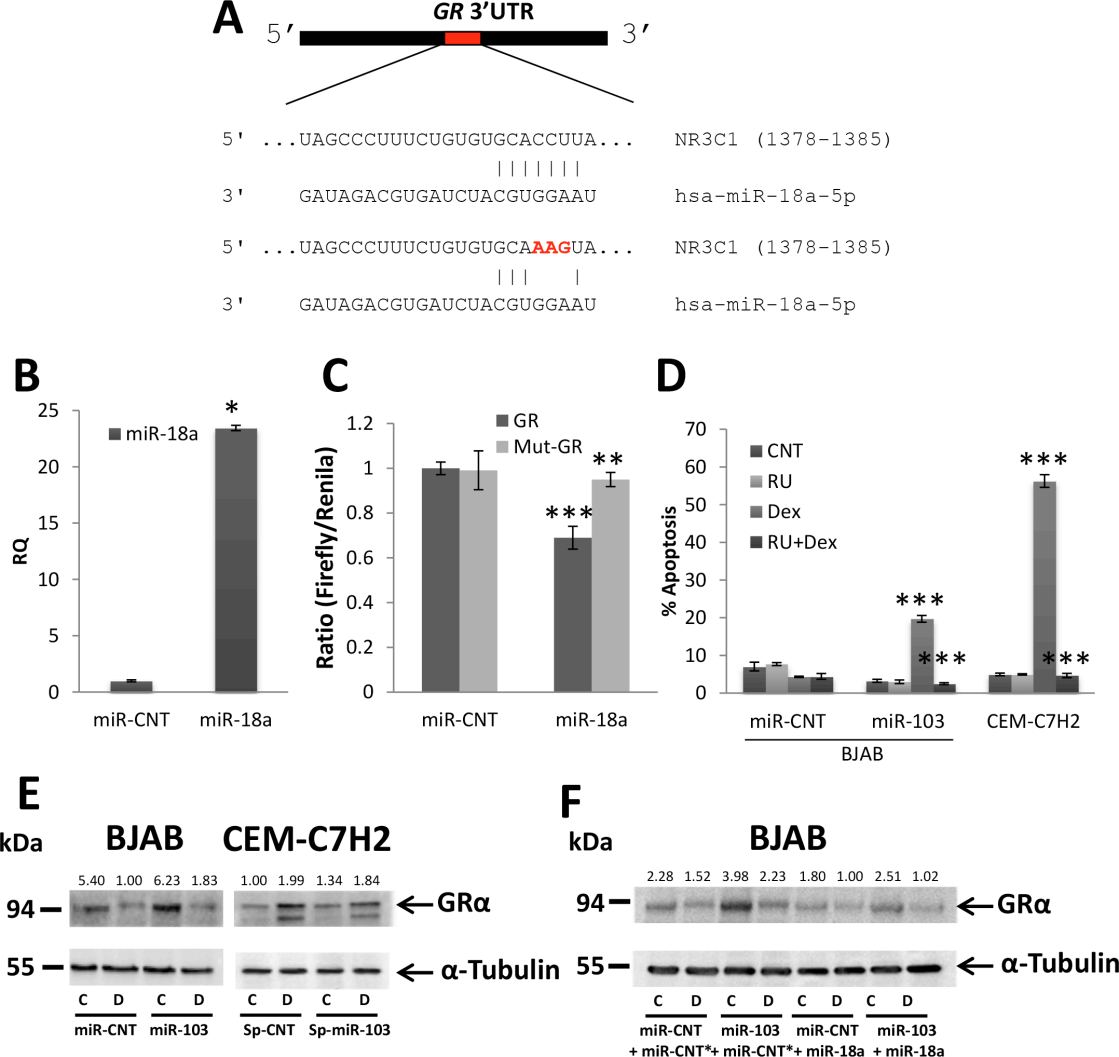
miR-103 upregulates GR via miR-18a downregulation (**A**) Schematic representation of the predicted binding sites for miR-18a in the 3′UTR of GR. (**B**) qRT-PCR for the expression of miR-103 in 293T cells transfected with either a control miR (miR-CNT) or miR-18a. (**C**) Wild type or mutated GR 3′UTR (GR or mut-GR, respectively) were fused to CDS of Firefly luciferase and co-transfected with Renilla luciferase into 293T cells overexpressing miR-18a or miR-CNT. The luciferase activity was measured and normalized to Renilla activity. (**D**) miR-103 overexpressing BJAB and CEM-C7H2 cells were treated with Dex and/or RU486 for 72 hrs. Percent apoptosis was determined by PI staining and flow cytometry analysis. Relative quantification of miR-18a and GR in untreated or Dex treated cells. (**E**) Western blot analysis of GRα in miR-103 overexpressing BJAB cells and miR-103 sponged CEM-C7H2 cells treated with Dex. (**F**) Western blot analysis of GRα in miR-103 overexpressing cells transfected with miR-18a and treated with Dex. C = Untreated, D = Dex-treated.

In order to decipher the mode by which miR-103-induced downregulation of miR-18a and miR-20a facilitates GCIA, we searched for target genes of the aforementioned miRs. Computational analysis (miRanda software) revealed that miR-18a possesses a 8mer base pair sequence compatible with the 3′UTR region of GR mRNA (*NR3C1*) (Figure 6A). To demonstrate that miR-18a indeed targets the 3′UTR of GR, we performed a luciferase assay. To this end, miR-18a overexpressing 293T cells (Figure 6B) were transfected with either the wild type or seed mutated 3′UTR of GR (Figure 6A). Luciferase activity was significantly reduced in the presence of miR-18a, while in the presence of the mutated 3′UTR of GR luciferase activity was restored, confirming that miR-18a directly targets the 3′UTR of GR (Figure 6C).

It was previously reported that GR is upregulated in GC-sensitive cells but downregulated in GC-resistant cells upon GC-treatment [15]. However, we found that GR is indeed upregulated by Dex treatment in GC-sensitive CEM-C7H2 but downregulated in GC-sensitive LOUCY and DAUDI and in all GC-resistant cells tested (Figure 6E and Supplementary Figure S4C). At the mRNA level, GR is upregulated only in the GC-sensitive CEM-C7H2 cells but remains unchanged in the rest GC-sensitive and GC-resistant cells (Supplementary Figure S4B). These results suggest that GR expression is regulated post-transcriptionally. Dex-induced GR mRNA upregulation in CEM-C7H2 correlates with miR-18a downregulation (Supplementary Figure S4A–S4B), whereas when GR level was unaffected by Dex, the level of miR-18a also remained unchanged (Supplementary Figure S4A–S4B), thus suggesting that miR-18a controls the stability of GR mRNA. miR-103 overexpression elicits both GR protein and mRNA upregulation (Figure 6E and Supplementary Figure S4C–S4D). Furthermore, sponging of miR-103 in CEM-C7H2 cells reduced the Dex-induced GR upregulation (Figure 6E), suggesting that GR is auto-upregulated, in part, by miR-103 upregulation. In contrast to GR mRNA, which was augmented following Dex treatment in miR-103 overexpressing cells (Supplementary Figure S4D), GR protein level was reversed by Dex treatment in both GC-resistant and GC-sensitive LOUCY and DAUDI cells (Figure 6E and Supplementary Figure S4C). However, although GR is eventually downregulated by Dex, in miR-103 overexpressing cells, still its level is mostly higher than miR-CNT cells treated with Dex. In addition, its basal level is the critical factor in advancing GCIA, since GCIA is still detectable (Figure 3F and Supplementary Figure S2A–S2E).

To reinforce the surmise that miR-103 downregulates miR-18a and upregulates GR by decreasing the level of c-Myc, we overexpressed c-Myc in miR-103 transfected cells. Indeed, c-Myc overexpression reversed the effect of miR-103 on miR-18a and GR expression (Supplementary Figure S4E–S4F). In order to confirm that miR-103-induced upregulation of GR is prompted by miR-18a downregulation, we restored miR-18a expression in miR-103 transfected cells. This manipulation blocked the upregulation of GR mediated by miR-103 (Figure 6F). The data suggest that GCIA induced by miR-103 is mediated, in part, by downregulation of c-Myc, which is followed by miR-18a downregulation and GR upregulation. Indeed, inhibition of GR by RU486 prevented GCIA in GC-sensitive CEM-C7H2 or in GC-resistant BJAB cells sensitized by transfection with miR-103 (Figure 6D).

### Suppression of miR-20a by miR-103 enables BIM upregulation

**Figure 7:**
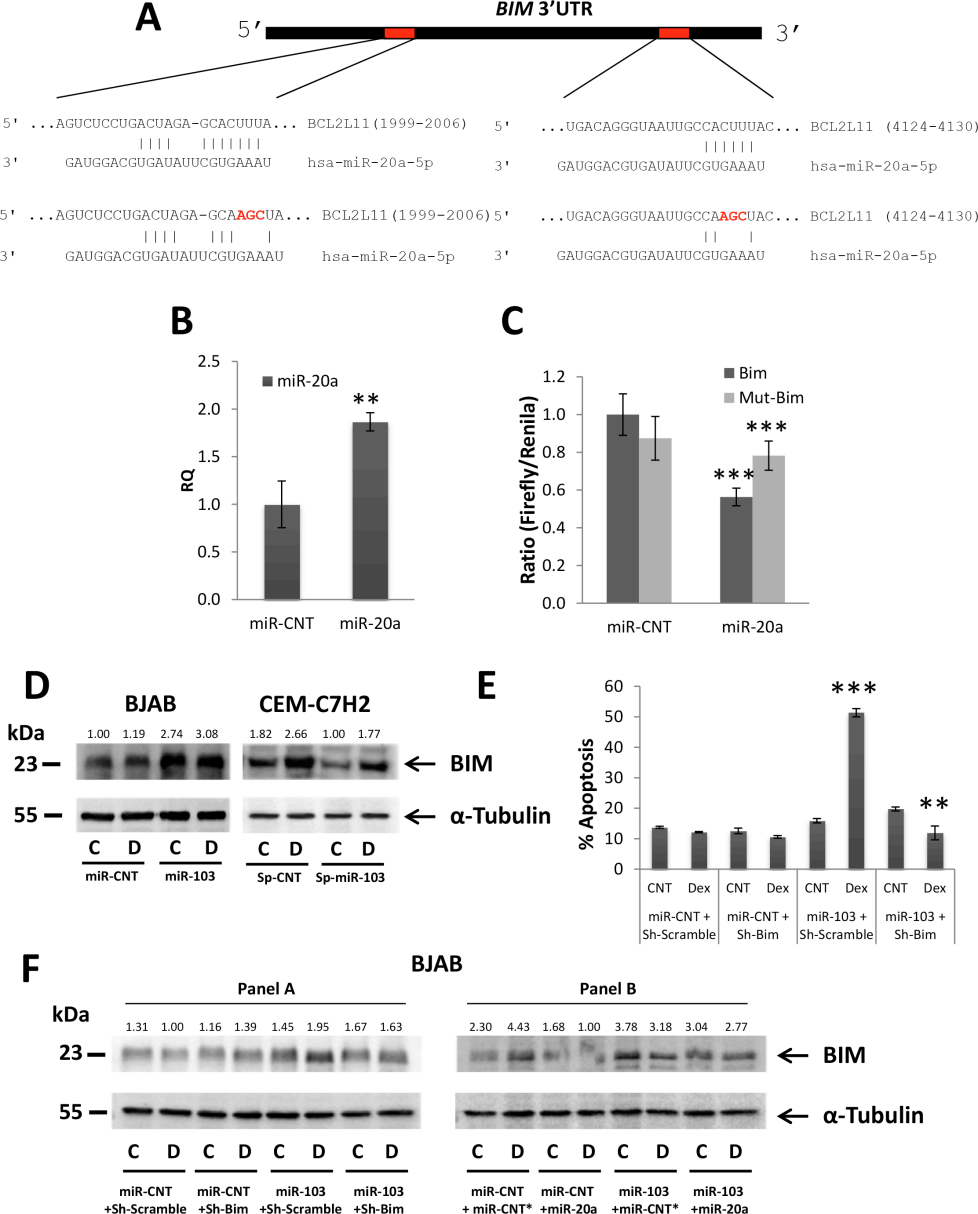
miR-103 upregulates BIM via miR-20a downregulation (**A**) Schematic representation of the two conserved binding sites for miR-20a in the 3′UTR of BIM mRNA. (**B**) qRT-PCR for the expression of miR-103 in 293T cells transfected with either a control miR (miR-CNT) or miR-20a. (**C**) Wild type or mutated BIM 3′UTR (BIM or mut-BIM, respectively) were fused to CDS of Firefly luciferase, and co-transfected with Renilla luciferase into 293T cells overexpressing miR-20a or miR-CNT. The luciferase activity was measured and normalized to Renilla activity. (**D**) Western blot analysis of Dex-treated miR-103 overexpressing BJAB and miR-103 sponged CEM-C7H2 using anti-BIM antibody. (**E**) miR-103 overexpressing BJAB cells were transfected with Sh-BIM and treated with Dex for 72 hrs. Percent of apoptotic cells was determined by PI staining. (**F**) Western blot analysis of BIMEL in miR-103 overexpressing BJAB cells transfected with Sh-BIM and treated with Dex (Panel A) in miR-103 overexpressing BJAB cells which were transfected with miR-20a and treated with Dex (Panel B). C = Untreated, D = Dex-treated.

Another function of miR-103 is to downregulate miR-20a (Figure 5B) which partakes in advancing GCIA (Figure 5C). We have therefore searched for potential targets of miR-20a that are involved in GCIA. We found (using miRanda software) that miR-20a contains two conserved sites compatible with the 3′UTR of BIM mRNA (Figure 7A). Luciferase assay of miR-20a overexpressing 293T cells (Figure 7B) transfected either with the predicted wild type or seed mutated 3′UTRs of BIM, confirms that miR-20a effectively targets the 3′UTRs of BIM, as indicated by the reduction in luciferase activity (Figure 7C). Partial restoration of luciferase activity in the presence of the mutated 3′UTRs of BIM further supports this finding (Figure 7C).

Indeed, BIM and miR-20a are reciprocally regulated in GC-sensitive CEM-C7H2 cells: while BIM mRNA expression is upregulated by Dex (Supplementary Figure S5B) [10], miR-20a is concomitantly downregulated (Supplementary Figure S5A). Interestingly, although miR-20a did not significantly change in both GC-resistant and GC-sensitive LOUCY and DAUDI cells (Supplementary Figure S5A), BIM was still upregulated significantly in the GC-sensitive LOUCY and DAUDI cells (Supplementary Figure S5B), suggesting that Dex induces BIM upregulation in these cells by a miR-103-independent mechanism. Indeed, although LOUCY and DAUDI are sensitive to GCIA, miR-103 is not upregulated by Dex in these cells, as in CEM-C7H2.

To establish a link between miR-103-induced miR-20a downregulation and BIM upregulation, we first looked for the effect of miR-103 overexpression on BIM. Indeed, miR-103 induced BIM upregulation both at the protein and mRNA levels, an activity that was augmented by Dex (Figure 7D and Supplementary Figure S5C–S5D). Furthermore, sponging miR-103 in CEM-C7H2 significantly reduced both basal and Dex-induced BIM levels (Figure 7D). The role of BIM upregulation in miR-103 mediated GCIA was verified with sh-RNA studies. Downregulating BIM expression abrogates miR-103 sensitization to GCIA (Figure 7E). Indeed, Sh-BIM reduced BIM expression to its basal level as in non-miR-103 transfected cells (Figure 7F panel A).

In order to establish that miR-103 upregulates BIM expression by decreasing the level of c-Myc, we overexpressed c-Myc in miR-103 transfected cells and observed reversal of the miR-103 effect on BIM expression (Supplementary Figure S5E). Finally, we demonstrated that miR-103-induced BIM upregulation is effectuated via the downregulation of miR-20a. While the level of BIM is upregulated upon miR-103 transfection, it is re-suppressed when miR-20a expression is restored (Figure 7F panel B).

## DISCUSSION

The present study unveils the molecular basis underlying GC-sensitivity in leukemic cells. Deep sequencing analysis points toward miR-103 as a potent effector in this molecular machinery. While miR-103 plays an essential role in some GC-sensitive cells, its ectopic expression in GC-resistant cells confers a GC-sensitive phenotype. Both inhibition of cellular proliferation and apoptosis are the hallmarks of effective GC-based therapy. Therefore, we focused our investigation on the molecular pathway by which miR-103 affects proliferation and apoptosis in response to GC.

miR-103 is encoded within the intron of the *PANK3* gene [39]. *PANK3* catalyzes phosphorylation of Pantothenate [43], which is a crucial step in the synthesis of Co-enzyme A [44] and activation of multiple metabolic enzymes, none of which is involved in proliferation and apoptosis [45]. It is therefore unlikely that miR-103 expression is solely controlled by transcription factors of *PANK3*. Indeed, about 30% of intronic miRNAs are transcribed independently of their host gene [46]. Our study indicates a miscorrelation between the basal expression levels of *PANK3* and miR-103. Nevertheless, upon GC stimulation, miR-103 and *PANK3* are co-regulated. We discovered that the *PANK3* promoter is activated by GR due to a GRE which we detected ~10,000 bp upstream to its coding region. Presumably, the GRE within the *PANK3* locus acts as an enhancer that upregulates miR-103 expression by nuclear GR.

miR-103 has controversial role in cancer development. On the one hand, miR-103 functions as a tumor suppressor. For example, it is downregulated in prostate cancer and by that virtue enables upregulation of its oncogenic target PDCD10, which promotes proliferation and cellular migration [47]. Furthermore, miR-103 reduces the proliferation of neuroblastoma cells and promotes their differentiation [48]. In addition, miR-103 suppresses proliferation of non-malignant cells as osteoblasts [49] and mouse intestinal crypt cells [38]. Indeed, IGF-1 stimulation of mouse intestinal crypt cells is accompanied by the downregulation of miR-103, which is followed by upregulation of its target genes: CCNE1, CDK2 and CREB1 [38]. On the other hand, miR-103 functions as an oncogene in some other cancerous cells. For example, it promotes cellular proliferation and inhibits apoptosis in hepatocellular carcinoma cells [50]. Furthermore, miR-103 is upregulated in colorectal cancer (serves as a marker for poor prognosis) and promotes tumor cell proliferation, migration and metastasis. In addition, miR-103 inhibits apoptosis, partially by targeting DICER and PTEN (in relation to proliferation and migration) [51], by targeting PER3 (in relation to apoptosis) [52] and by targeting DAPK and KLF4 (in relation to metastasis) [53]. miR-103 also targets metalloproteinase 3 and mediates endometrial cancer cells growth and invasion [54]. The debatable role of miR-103 in various cancer types points to tissues specific effect.

We found that miR-103 expression is lower in bone marrow-derived MCs of ALL patients than in healthy bone marrow. Therefore, we suggest that miR-103 downregulation might be essential for ALL development and acts as a tumor suppressor. Indeed, proliferation induction of normal PBMCs, which was accompanied by miR-103 downregulation, confirms this concept. GCs are known to reduce proliferation of many cell types [36, 55–57]. This effect has been attributed, in part, to a decreased activity of CDK2 and CDK4, decreased levels of cyclin D, E2F and c-Myc, and increased levels of the CDK inhibitor p21^Cip1 [57]^. These data are corroborated by our findings that Dex significantly reduces proliferation of GC-sensitive cells while in GC-resistant cells, miR-103 overexpression imitates the GC effect. This observation suggests that the GC-mediated inhibition of cellular proliferation is followed, in part, by miR-103 upregulation. Remarkably, miR-103 inhibits CDK2 and cyclin E1 in ALL and Burkitt's lymphoma cells as in the mouse intestinal crypt [38].

Screening in prediction software failed to detect proteins belonging to the apoptotic signal of GC as direct targets of miR-103. However, we found that c-Myc, which is essential for GCIA [21], is a central player in miR-103-mediated GCIA. c-Myc is an onco-protein deregulated in a wide-range of tumors [58]. Genomic rearrangements associated with c-Myc correlate with poor prognosis in diffuse large B cell lymphoma patients undergoing R-CHOP chemotherapy [59]. Hence, c-Myc upregulation may be a cause for GC-resistance, thus proposing that its downregulation by miR-103 is an essential step in advancing GCIA. While c-Myc is not a target of miR-103, we identified two c-Myc activators, c-Myb and DVL1, as directmiR-103 targets. c-Myb is a leucine zipper transcription factor essential for hematopoiesis, especially of the T-cell lineage [42]. Its expression in T-ALL and AML is sometimes affected by chromosomal aberrations and amplifications [60]. c-Myb directly mediates c-Myc transcription by virtue of its binding to the c-Myc promoter [61]. Also, DVL1, whose triggering occurs via the Wnt signaling pathway [62], activates c-Myc indirectly by stabilizing β-Catenin [63], which acts as c-Myc transcription factor [40]. In T-ALL, both c-Myb and β-Catenin are activated. By contrast, in Burkitt's lymphoma, c-Myb is poorly expressed and therefore miR-103 effectuates c-Myc downregulation via targeting of DVL1.

Among the many genes affected by c-Myc is miR-17~92a [34], which is upregulated in many human cancers [64]. In analyzing the function of each miR-17~92a member we observed that even thought all six miRNAs are downregulated by miR-103, only miR-18a and miR-20a affect GCIA. Indeed, we demonstrated that upregulation of GR by miR-103 is induced via downregulation of both c-Myc and miR-18a, whereas BIM upregulation is induced via the downregulation of both c-Myc and miR-20a.

GR expression level is auto-upregulated in most of the GC-sensitive cells as it binds to its own promoter [12]. However, when miR-103 is sponged, GR is still auto-upregulated but to a lower extent. Hence, GR upregulation by GC proceeds along two different pathways: 1) GR binding to its GRE, 2) GR induced miR-103 upregulation, which is followed by c-Myc and miR-18a downregulation that leads to GR mRNA stabilization. When GC-resistant cells are treated by the hormone, GR is not upregulated and even downregulated as a consequence of proteasomal degradation [65, 66]. Upregulation of miR-103 in such cells increases GR expression but does not protect it from GC-induced degradation by the proteasome. Albeit the marked reduction in GR expression, miR-103 still activates GCIA, thus suggesting that a basal level of the GR is sufficient to enable a GC-mediated apoptotic response. GR phosphorylation after GC binding is an additional level of GR regulation. Indeed, our preliminary data suggest that miR-103 affects GR phosphorylation on both activatory and inhibitory phosphorylation sites (data not shown). It is therefore difficult to assess the consequences of miR-103-mediated GR phosphorylation, its effect on GR functionality and its subsequent contribution to GCIA.

Wang et al. [10] demonstrated that BIM is upregulated by GC and its ablation abrogates GCIA. However, BIM promoter does not contain GRE, suggesting an indirect mechanism for its upregulation. Indeed, it was previously demonstrated that miR-17~92a downregulates BIM expression, but only miR-92a, miR-19 and miR-17 were shown to be involved in this outcome [30, 32, 33]. In our hands, miR-20a downregulation by GC is responsible for both BIM upregulation and GCIA while other members of miR-17~92a do not affect BIM expression.

**Figure 8:**
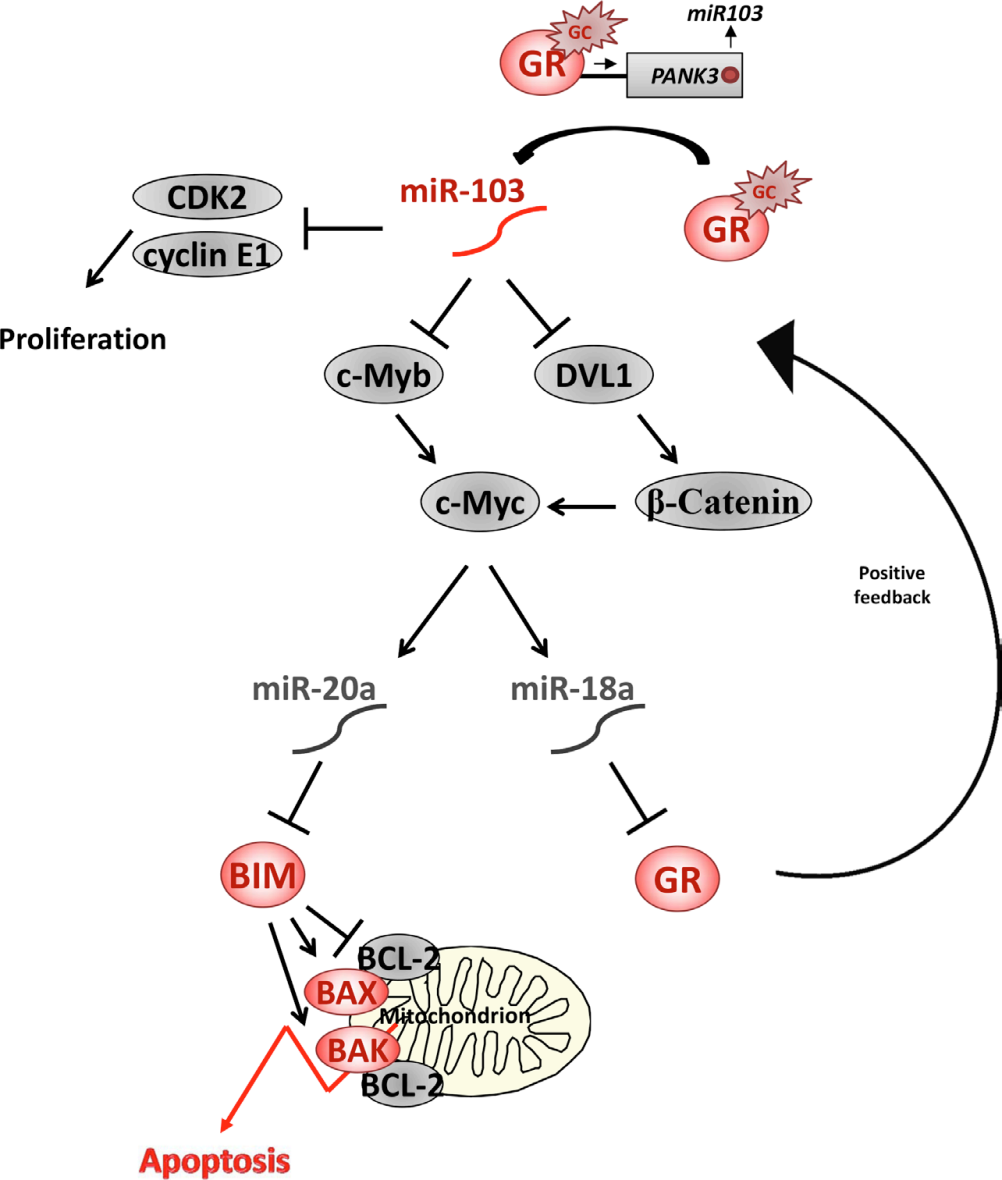
A model of miR-103 network in GCIA GC upregulates miR-103 expression by direct binding of activated GR to a GRE sequence in the promoter of its host gene PANK3. miR-103 inhibits CDK2 and Cyclin E1 translation thus reducing cellular proliferation. In addition, miR-103 inhibits c-Myb and/or DVL1 by binding to their 3′UTR. c-Myb downregulation results with c-Myc transcription inhibition, whereas DVL1 downregulation results with β-Catenin degradation and transcription inhibition of c-Myc. c-Myc ablation is followed by downregulation of miR-18a and miR-20a. As miR-18a inhibits GR translation, expression of GR is upregulated. GR accumulation accelerates the response to GC and further elevates miR-103 in a positive feedback pathway. miR-20a inhibits BIM translation and, therefore, miR-103-induced miR-20a downregulation is followed by BIM upregulation. Pro-apoptotic BIM activates the mitochondrial apoptotic pathway by it inhibitory interaction with Bcl-2 proteins, which is followed by BAK and BAK oligomerization, release of cytochrom C and SMAC/Diablo, thus leading to GCIA [72].

Based on the data presented herein, we propose a model depicted in Figure 8. c-Myc deregulation in leukemic cells upregulates miR-20a and miR-18a, which are potential inhibitors of GCIA. Upon GC treatment, miR-103 is activated in some GC-sensitive cells and targets c-Myb and/or DVL1. Consequently, c-Myc is downregulated, thus blocking miR-20a and miR-18a. This outcome enables BIM expression and GR auto-upregulation with eventual activation of the mitochondrial apoptotic pathway. Since CDK2 and cyclin E1 induce cellular proliferation, their inhibition by miR-103 is also instrumental in blocking cell division. This model does not imply that miR-103 upregulation is mandatory for GCIA since there are alternative mechanisms by which BIM expression is upregulated and initiates the mitochondrial apoptotic pathway. However, conferring miR-103 expression in GC-resistant cells facilitates the GCIA process, marking miR-103 as a potential molecule for therapeutic intervention in ALL.

The present study highlights the possibility of relieving the GC-resistance therapeutic obstacle by manipulating miR-103 levels in leukemia cells amenable to GC based therapy. Exogenic insertion of miRNAs into mammalian cells is not yet applicable for clinical practice. However, miRNA delivery to cells is under extensive research, which may open new opportunities for using these molecules as therapeutic drugs.

## MATERIALS AND METHODS

### Cells and reagents

CEM-C7H2 and CUTLL are human T-ALL cell lines [67, 68]. MOLT-4, Loucy, BJAB, SUD-H6 and DAUDI cell lines were obtained from ATCC. Dex, RU486, Propidium iodide (PI), puromycin and Tri-reagent were purchased from sigma and TransIT-LT1 from Mirus. Bone marrow cells were collected from normal donors and ALL patients. All experiments with human cells were approved by an institutional Helsinki committee and the Ministry of Health (0676-13-HMO).

### Deep sequencing

RNA of cell samples was extracted by Tri-reagent. 1 μg of each sample was sequenced on an Illumina Genome Analyzer [68]. Data analysis was performed by a miRNAkey application [69].

### miRNAs expression plasmids

Sh-scramble-pLKO.1 control plasmid, was described earlier [70]. miR-CNT plasmid refers to miR-K2 and SP-CNT plasmid refers to Sp-UL112, they were provided by Dr. Dafna Nachmani [71]. Sh-c-Myc and sh-BIM with their control plasmids were purchased from Openbiosystem and miR-17~92a plasmids from GeneCopoeia.

### miRNA overexpression

The miRNA oligomers used in this study are outlined in Supplementary Table S3. Oligos were annealed, phosphorylated and inserted into a pTER plasmid in order to connect the overexpressed miRNA to the U6 promoter. The reconstructed pTER was sequenced by the H1 primer 5′ CGCTGACGTCATCAACCCGC 3′. The miRNA/U6 construct was excised and inserted into a Sin-GFP plasmid.

### miRNA sponge

The oligonucleotides of miRNA sponges are outlined in Supplementary Table S4. These oligos were annealed, phosphorylated and inserted one after the other into a pBSII plasmid. The reconstructed pBSII plasmid was sequenced by the T7 primer 5′ TAATACGACTCACTATAGGG 3′. Consequently, the joint pairs A and B were excised and inserted into a Sin-GFP plasmid.

### Stable transfection

Lentivirus particles were prepared in 293T cells with the pCMV/Δ8.91 packaging vector and pMD2-VSV-G envelope construct by using TransIT-LT1 transfection reagent. Lentivirus supernatant was collected after 48 hrs and incubated with cells for 24 hrs. pLKO.1, pEZX and pGIPZ transfected cells were enriched by selection in 1–4 μg/ml puromycin.

### Apoptosis

Cells were treated with 100 nM Dex for 72 hrs. Percent apoptotic cells was determined by PI-uptake as measured in a FACS Calibur flow cytometer (BD).

### Cellular proliferation

A BrdU Cell Proliferation Assay kit (Exalpha Biological, Inc) or a BrdU Flow Kit (BD Pharmingen) were used to determine cellular proliferation.

### Western blots and antibodies

Total lysate of 10^6^ cells was prepared in 50 μl protein sample buffer (PSB) x1.5. The following antibodies were used for immuno-blotting: anti-β-Catenin and anti-GR (BD Transduction Laboratories); anti-BIM (Calbichem); anti-CDK2, anti-c-Myb, anti-DVL1 and anti-c-Myc (Santa Cruz Biotechnology); anti-cycline E1 (Cell Signaling Technology); and anti-α-Tubulin (Sigma). Immune-blots images are presented by Image Lab 3.0 software. Specific bands were cropped by transferring images to Adobe Photoshop CS5.

### qRT-PCR

Total RNA was isolated using miRNeasy mini kit (QIAGEN). c-DNA was prepared by Taqman MicroRNA Reverse Transcription Kit (Applied Biosystem). q-PCR was performed using TaqMan 2X Universal PCR Master Mix (Applied Biosystem) with the corresponding custom primers.

### CFSE staining and T cell proliferation

PBMCs were extracted by using Ficoll gradient (Lymphoprep), stained for CFSE at a final concentration of 0.5 μM at 37°C for 15 min and undergo T-cell activation by using anti-CD3 (OKT3 eBioscience at a final concentration of 300 ng/mL) and anti-CD28 (BD, at a final concentration of 1 μg/mL) antibodies. anti-CD3 was bound to the plate for 1 hour in 37°C before seeding the cells and anti-CD28 was using solubility. PBMCs were then incubated for 4 days at 37°C and 5% CO_2_, stained for CD3 and was read by flow cytometry.

### Chromatin IP

The EpiTech ChIP OneDay kit (Qiagen) was employed. IP antibodies used were: Rabbit-IgG (Qiagen), anti-GR (cell signaling) and anti-H3K4me1 (abcam). qRT-PCR was performed by syber green enzyme with the following primers: *PANK3*-GRE, GAPDH positive control and MYOD1 negative control (Qiagen). GR-GRE primers were planned as follow: Fw – 5′ ATTCTT GTGCCTATGCAGACATTT 3′ and 5′ TGAATGCGTG CATATTCACACTA 3′. % Enrichment was calculated according to the formula: 2^(C_T_ Mean(Input) - C_T_ Mean of (Interested fraction))*100.

### Luciferase assay

The wild type and mutated 3′UTR of DVL-1, c-Myb, GR and Bim were ordered from Syntezza Bioscience IDT company into PUC57 vector. 3′UTRs were excised from PUC57 vector using XBA1 restriction sites and then cloned into a Firefly luciferase reporter PGL3 vector. The inserts and their proper orientation were confirmed by sequencing. For the right orientation we used the following primer: GAGTTGTGTTTGTGGACGAA. For validating the correct sequence of both wild type and mutated 3′UTRs, we used the following primers: c-Myb: CCATGTGACATTTAATCCAGATTG, BIM: first site-GAGTTGTGTTTGTGGACGAA and second site- ATCCCTGCTGATTTAGCC, GR: TACACATCCCTAAT GTGTGC. 293T cells were plated in 24-well plates and 24 hrs later were transfected with 200 ng of a Firefly luciferase reporter vector and 50 ng of the control Renilla luciferase pRL-CMV (Promega) using the LT1 transfection reagent (Mirus). Firefly and Renilla luciferase activities were measured consecutively with the Dual-Luciferase Assay System (Promega), 48 hrs following transfection. Firefly luciferase activity was normalized to Renilla luciferase activity and then normalized to the average activity of the control reporter.

### Statistical analysis

Each experiment was repeated at least three times. *P-value* was calculated using the Fisher-Irwin test. *P*-values marking: *p-Value < 0.05, **p-Value < 0.01 and ***p-Value < 0.001. *P*-values below 0.05 were considered statistically significant.

## SUPPLEMENTARY MATERIALS







## Abbreviations

GC: Glucocorticoid
GCIA: GC-induced apoptosis
CDK2: Cyclin dependent kinase
GR: GC receptor
GRE: GC response element
PRED: Prednisone
ALL: Acute lymphoblastic leukemia
PGR: Prednisone good responder
PPR: Prednisone poor responder
Dex: Dexamethasone
PI: Propidium iodide
MC: mononuclear cells
ND: normal donor
UTR: untranslated region
RQ: relative quantification
ChIP: chromatin immunoprecipitation
