# Supplementary material for: miR-103 inhibits proliferation and sensitizes hemopoietic tumor cells for glucocorticoid-induced apoptosis
Source: Oncotarget. 2016 Nov 18;8(1):472–89. doi: 10.18632/oncotarget.13447 (PMC5352135; doi:10.18632/oncotarget.13447)
Supplement: Supplementary file 1 [file oncotarget-08-472-s001.pdf]

# miR-103 inhibits proliferation and sensitizes hemopoietic tumor cells for glucocorticoid-induced apoptosis

## Supplementary Materials

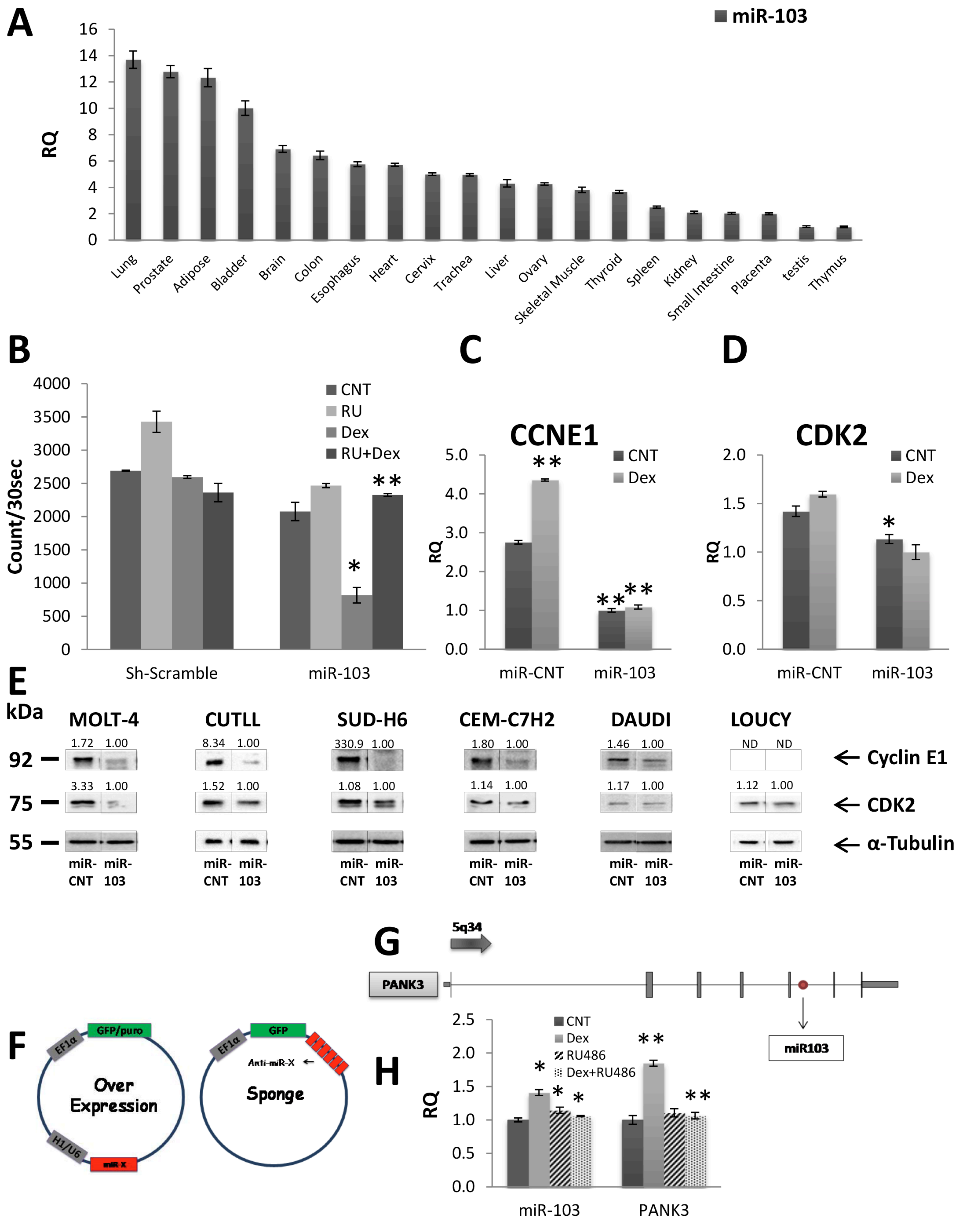


**Supplementary Figure S1: miR-103 inhibits cellular proliferation.** (**A**) Tissue-dependent miR-103 distribution. qRT-PCR analysis of miR-103 in human normal tissues. (**B**) miR-103 overexpressing BJAB cells were treated with Dex and/or RU486 for 72 hrs. Cell viability was determined by Flow cytometry. (**C**) and (**D**) Relative quantitation of Cyclin E1 (C) and CDK2 (D) in miR-103 overexpressing BJAB cells treated with Dex for 24 hrs. (**E**) miR-103 overexpressing GC-sensitive (DAUDI, CEM-C7H2) and –resistant cells (MOLT-4, CUTLL and SUD-H6) were treated with Dex for 24 hrs and then subjected to Western blot analysis using anti Cyclin E1 and CDK2 antibodies. C = Untreated, D = Dex-treated. (**F**) The *PANK3* gene is depicted with exons as length rectangles, introns as lines and UTRs as width rectangles. miR-103 is encoded in the intron between the fourth and fifth exons and marked red. (**G**) Relative quantitation of miR-103 and *PANK3* in CEM-C7H2 cells treated with Dex and/or the GR inhibitor RU486 by qRT-PCR. (**H**) miRNA overexpression and sponge plasmids. Overexpression of miRNAs with GFP as a marker is driven by a U6 promoter whereas puromycin resistance marker (pLKO) is driven by H1 promoter. Sponge plasmids were constructed with six repeats of an imperfect miRNA antisense in the 3′UTR of the GFP gene.

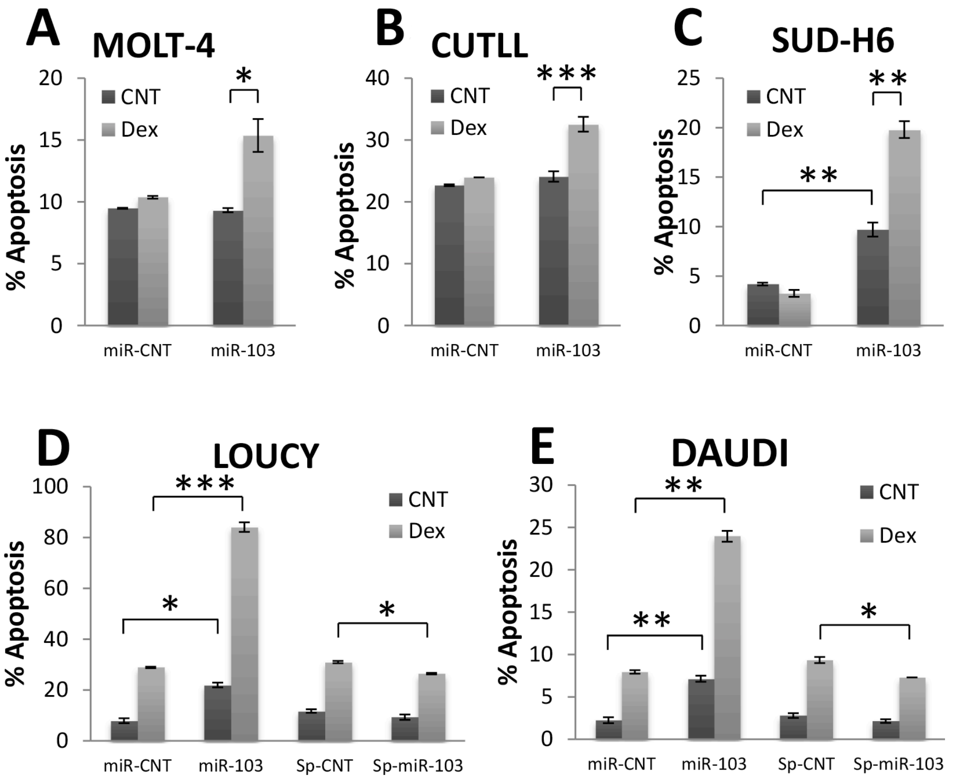


**Supplementary Figure S2:** (**A**–**E**) miR-103 sensitizes GC-resistant cells to GCIA. miR-103 overexpressing GC-resistant MOLT-4, CUTLL and SUD-H6 cells (A–C) and miR-103 overexpressing or sponged GC-sensitive LOUCY and DAUDI (D–E) were exposed to Dex for 72 hrs and the percent of PI-positive apoptotic cells was assessed by Flow cytometry.

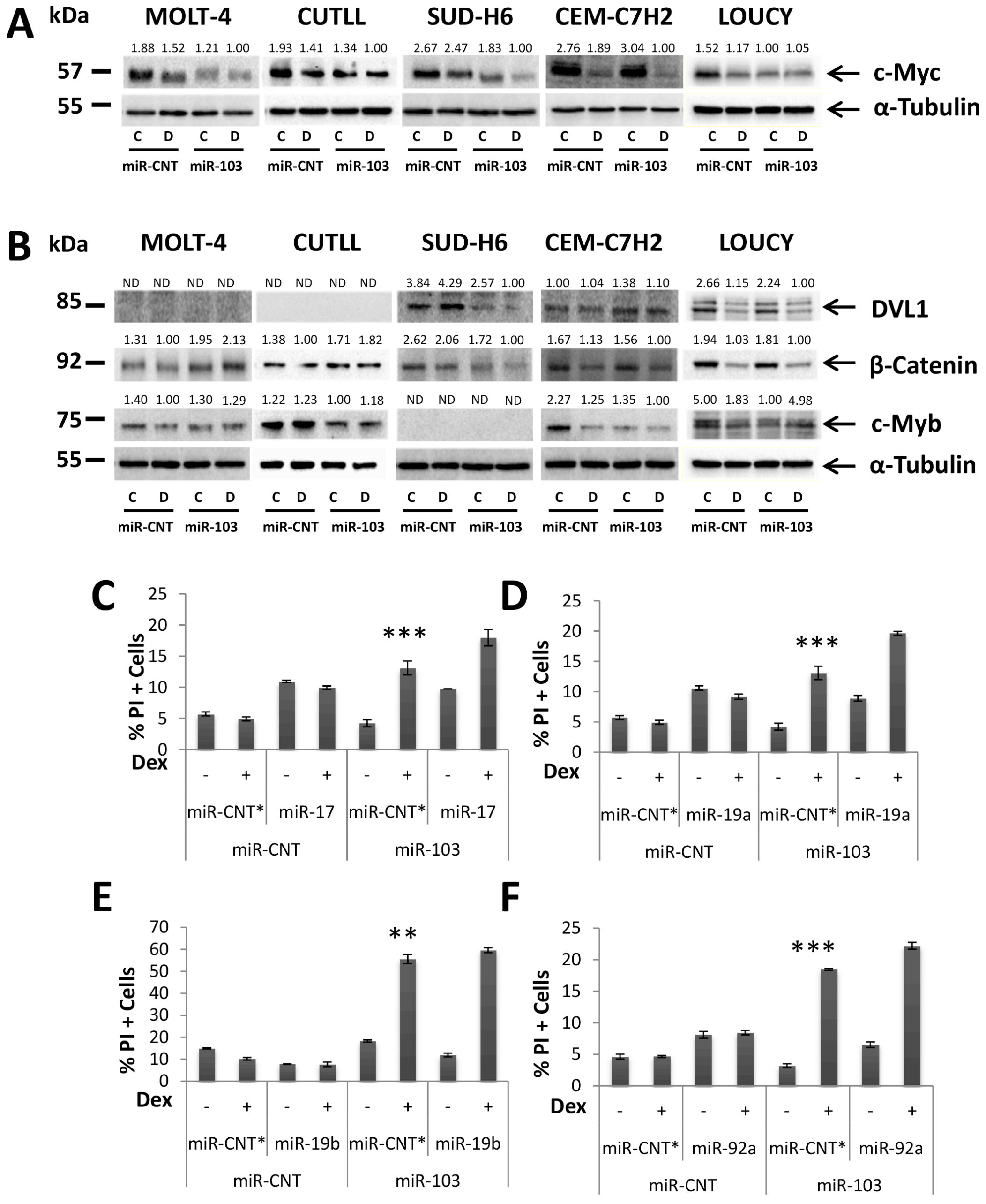


**Supplementary Figure S3:** (**A**) and (**B**) Western blot analysis of miR-103 overexpressing GC-resistant MOLT-4, CUTLL and SUD-H6 cells and GC-sensitive CEM-C7H2 and LOUCY cells using c-Myc (A) and DVL1, β-Catenin and c-Myb (B) antibodies**.** (**C**–**F**) miR-103 overexpressing BJAB cells were transfected with either miR-17 (C), miR-19a (D), miR-19b (E) or miR-92a (F). The cells were treated with Dex for 72 hrs and percent of apoptotic cells was determined by PI staining.

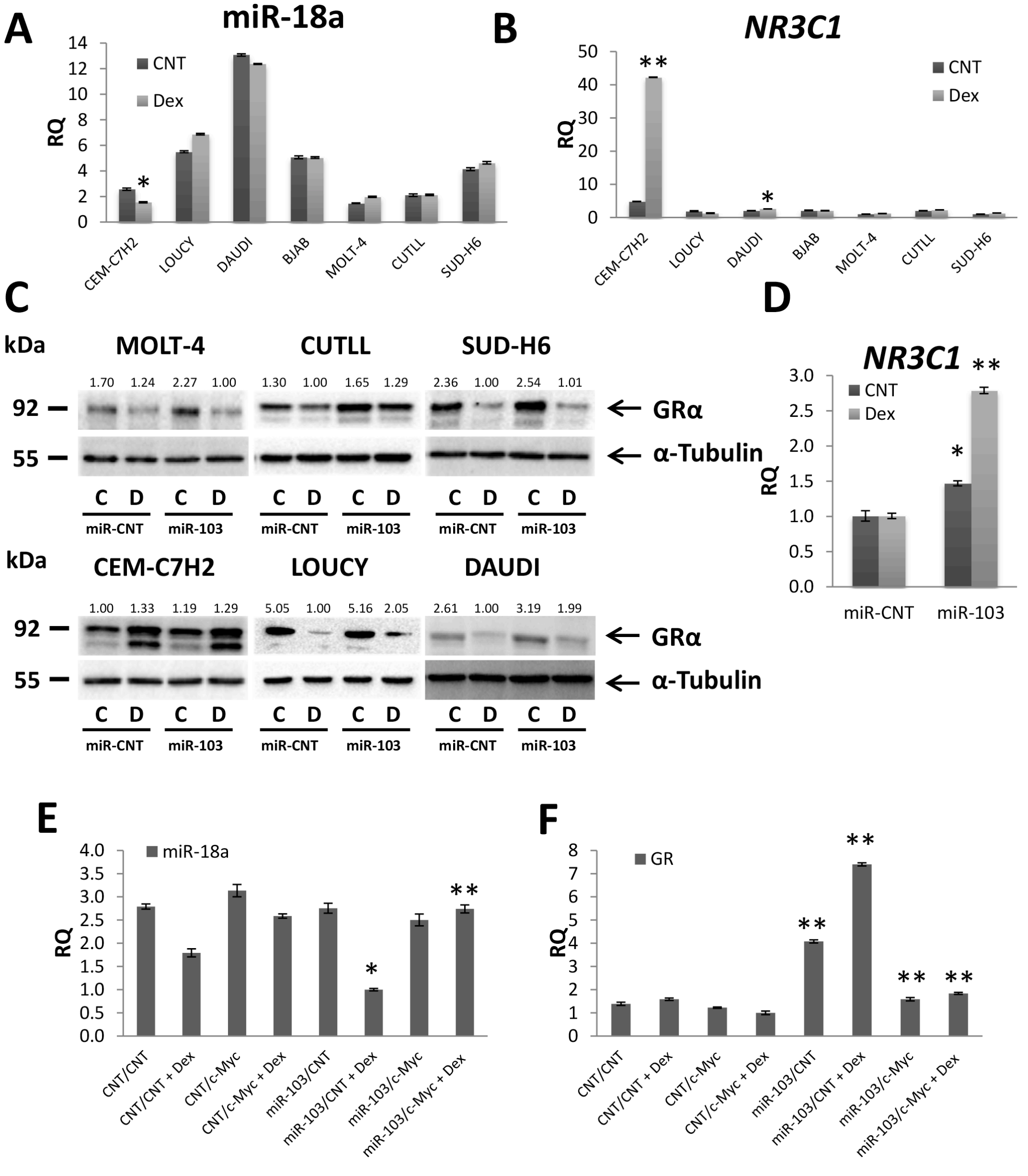


**Supplementary Figure S4:** (**A**) and (**B**) Relative quantification of miR-18a (A) and GR (NR3C1) mRNA (B) in GC-sensitive CEM-C7H2, LOUCY and DAUDI cells and GC-resistant BJAB, MOLT-4, CUTLL and SUD-H6 cells untreated or treated with Dex for 24 hrs. (**C**) Western blot analysis of miR-103 overexpressing MOLT-4, CUTLL and SUD-H6 GC-resistant cells (upper panel) and CEM-C7H2, LOUCY and DAUDI GC-sensitive cells (bottom panel) using anti-GR antibody. (**D**) qRT-PCR of GR (NR3C1) mRNA in miR-103 or miR-CNT overexpressing BJAB cells untreated or treated with Dex for 24 hrs. (**E**) and (**F**) Relative quantitation of miR-18a (E) and GR mRNA (F) in miR-103 or miR-CNT overexpressing BJAB cells transfected with c-Myc overexpression or CNT plasmids, and treated or untreated with Dex for 24 hrs.

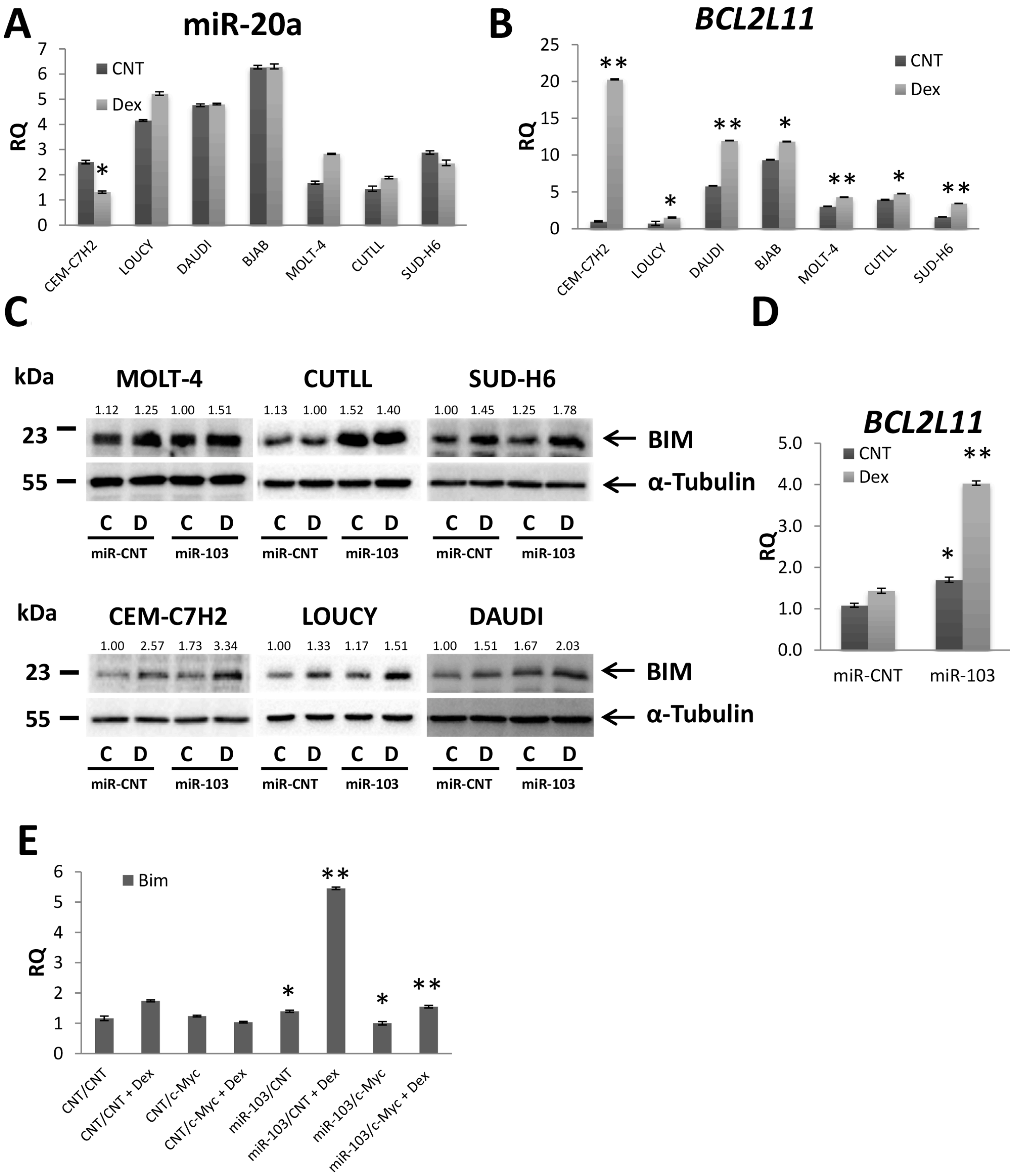


**Supplementary Figure S5:** (**A**) and (**B**) Relative quantification of miR-20a (A) and BIM (BCL2L11) mRNA (B) in GC-sensitive CEM-C7H2, LOUCY and DAUDI cells and GC-resistant BJAB, MOLT-4, CUTLL and SUD-H6 cells untreated or treated with Dex for 24 hrs. (**C**) Western blot analysis of miR-103 overexpressing MOLT-4, CUTLL and SUD-H6 GC-resistant cells (upper panel) and CEM-C7H2, LOUCY and DAUDI GC-sensitive cells (bottom panel) using anti-BIMEL antibody. (**D**) qRT-PCR of BIM (BCL2L11) mRNA in miR-103 or miR-CNT overexpressing BJAB cells untreated or treated with Dex for 24 hrs. (**E**) Relative quantitation of BIM mRNA in miR-103 or miR-CNT overexpressing BJAB cells transfected with c-Myc overexpression or CNT plasmids, and treated or untreated with Dex for 24 hrs.

**Supplementary Table S1: The output of deep sequencing analysis of untreated CEM-C7H2 versus CEM-C7H2 cells treated with Dex performed by miRNAkey application [69].** See Supplementary_Table_S1

**Supplementary Table S2: The output of deep sequencing analysis of untreated MOLT-4 versus MOLT-4 cells treated with Dex performed by miRNAkey application.** See Supplementary_Table_S2

**Supplementary Table S3: The oligonucleotide sequence used to construct miRNAs overexpression plasmids**

| Oligos to mi-RNAs over-expression constructs | | |
|---|---|---|
| has-miR-103 | FW | 5′GATCCCCAGCAGCATTGTACAGGGCTATGATTCAAGAGATCATAGCCCTG TACAATGCTGCTTTTTGGAAA 3′ |
| | RV | 5′AGCTTTTCCAAAAAAGCAGCATTGTACAGGGCTATGATCTCTTGAATCATAG CCCTGTACAATGCTGCTGGG 3′ |
| has-miR-30e | FW | 5′GATCCCCTGTAAACATCCTTGACTGGAAGTTCAAGAGACTTCCAGTCAAGG ATGTTTACATTTTTGGAAA 3′ |
| | RV | 5′AGCTTTTCCAAAAATGTAAACATCCTTGACTGGAAGTCTCTTGAACTTCCAG TCAAGGATGTTTACAGGG 3′ |
| has-miR-30d | FW | 5′GATCCCCTGTAAACATCCCCGACTGGAAGTTCAAGAGACTTCCAGTCGGGGA TGTTTACATTTTTGGAAA 3′ |
| | RV | 5′AGCTTTTCCAAAAATGTAAACATCCCCGACTGGAAGTCTCTTGAACTTCCAGT CGGGGATGTTTACAGGG 3′ |
| has-miR-19b | FW | 5′GATCCCCTGTGCAAATCCATGCAAAACTGATTCAAGAGATCAGTTTTGCATGGA TTTGCACATTTTTGGAAA 3′ |
| | RV | 5′AGCTTTTCCAAAAATGTGCAAATCCATGCAAAACTGATCTCTTGAATCAGTTTT GCATGGATTTGCACAGGG 3′ |
| has-miR-20a | FW | 5′GATCCCCTAAAGTGCTTATAGTGCAGGTAGTTCAAGAGACTACCTGCACTATAAGC ACTTTATTTTTGGAAA 3′ |
| | RV | 5′AGCTTTTCCAAAAATAAAGTGCTTATAGTGCAGGTAGTCTCTTGAACTACCTGCAC TATAAGCACTTTAGGG 3′ |
| has-miR-223 | FW | 5′GATCCCCTGTCAGTTTGTCAAATACCCCATTCAAGAGATGGGGTATTTGACAAACT GACATTTTTGGAAA 3′ |
| | RV | 5′AGCTTTTCCAAAAATGTCAGTTTGTCAAATACCCCATCTCTTGAATGGGGTATTTGA CAAACTGACAGGG 3′ |
| has-miR-15b | FW | 5′GATCCCCCGAATCATTATTTGCTGCTCTATTCAAGAGATAGAGCAGCAAATAATGAT TCGTTTTTGGAAA 3′ |
| | RV | 5′AGCTTTTCCAAAAACGAATCATTATTTGCTGCTCTATCTCTTGAATAGAGCAGCAAA TAATGATTCG GGG 3′ |
| has-miR-16 | FW | 5′GATCCCCTAGCAGCACGTAAATATTGGCGTTCAAGAGACGCCAATATTTACGTGCTGC TATTTTTGGAAA 3′ |
| | RV | 5′AGCTTTTCCAAAAATAGCAGCACGTAAATATTGGCGTCTCTTGAACGCCAATATTTA CGTGCTGCTAGGG 3′ |

**Supplementary Table S4: The oligonucleotide sequence used to construct miRNAs sponge plasmids**

**Oligos to mi-RNAs sponge construct**

| | | | |
|---|---|---|---|
| has-miR-103 | A | FW | 5′ TCGACTCATAGCCCTGTGTATGCTGCTAGAGTCATAGCCCTGTGTATGCTGCT AG AGTCATAGCCCTGTGTATGCTGCTA 3′ |
| | | RV | 5′AGCTTAGCAGCATACACAGGGCTATGACTCTAGCAGCATACACAGGGCTATG ACTCTA GCAGCATACACAGGGCTATGAG 3′ |
| | B | FW | 5′AGCTTTCATAGCCCTGTGTATGCTGCTAGAGTCATAGCCCTGTGTATGCTGCT AGAGTC ATAGCCCT GTGTATGCTGCTG 3′ |
| | | RV | 5′AATTCAGCAGCATACACAGGGCTATGACTCTAGCAGCATACACAGGGCTATG ACTCTAG CAGCATACACAGGGCTATGAA 3′ |
